# Activity Coefficients of HCl in Solutions Related to “Tris” Buffers in Artificial Seawater. I. HCl + TrisHCl + H_2_O from 1.0 to 5.0 mol kg^–1^ Ionic Strength, and from 5 to 45 °C

**DOI:** 10.1021/acs.jced.5c00035

**Published:** 2025-04-18

**Authors:** Igor Maksimov, Toshiaki Asakai, Yuya Hibino, Simon L. Clegg

**Affiliations:** † National Institute of Advanced Industrial Science and Technology (AIST), National Metrology Institute of Japan, 1-1-1 Umezono, Tsukuba, Ibaraki 305-8563, Japan; ‡ School of Environmental Sciences, University of East Anglia, Norwich NR4 7TJ, United Kingdom

## Abstract

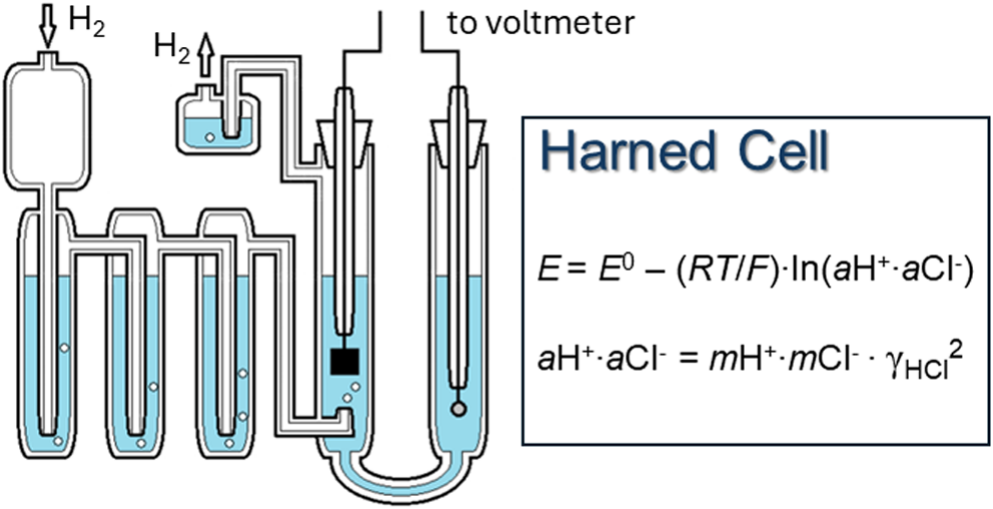

The substance Tris (2-amino-2-hydroxymethyl-1,3-propanediol, CAS 77-86-1), and its protonated form TrisH^+^, are used in the preparation of pH buffers in artificial seawater media for applications in marine chemistry. The development of a chemical speciation model of the buffer solutions has been proposed in order to quantify the effects of composition change on buffer pH and to address the metrological requirement for traceability of pH to SI base units. Such a model should be based upon data yielding solvent activities and mean activity coefficients (especially those of H^+^ with conjugate anions such as Cl^–^ or SO_4_^2–^) for aqueous solutions of single solutes (e.g., HCl, TrisHCl) and simple mixtures over a temperature range of about 0 to 40 °C. There are currently few data for solutions containing the ion TrisH^+^, and these are mostly restricted to 25 °C. Here, in the first of a series of studies, we present Harned cell measurements of potentials in solutions containing HCl and TrisHCl from 5 to 45 °C, yielding mean activity coefficients of HCl. The results at 25 °C are found to agree closely with those of literature studies. The Harned cell technique is described in detail, including the preparation of electrodes.

## 1 Introduction

The seawater total hydrogen ion pH scale, which is used for the most accurate measurements of ocean pH, was established from measurements of cell potentials of solutions of artificial seawater acidified with HCl, and of buffer solutions containing equimolal Tris and its conjugate acid TrisH^+^.^1^ The calibration of the scale, for solution compositions corresponding to the same ratios of major ions as found in normal ocean water, is still largely limited to salinities above 20 for reasons given by Clegg et al.^2^ Artificial seawaters used in the preparation of total pH buffers have compositions such as that listed in Table 1. About 90 mol % consists of Na^+^ and Cl^–^ ions, and there are much smaller amounts of Mg^2+^, SO_4_^2–^, Ca^2+^, and K^+^. The minor species present in natural seawater^3^ are omitted as they are not expected to influence the activity coefficients of other solutes (because their molalities are too low).

**Table 1:** Typical Composition of Artificial Seawater^a^

solute species	molality (at salinity 35)	mol %
Na^+^	0.48618	41.89
Mg^2+^	0.05474	4.716
Ca^2+^	0.01075	0.926
K^+^	0.01058	0.912
Cl^–^	0.5692	49.04
SO_4_^2–^	0.02927	2.522

a From DelValls and Dickson.^1^ In Tris buffer solutions cation TrisH^+^ (generally 0.04 molal or lower) substitutes for an equal molality of Na^+^, and the same molality of neutral solute Tris is added.

Dickson et al.^4^ and later Clegg et al.^2^ have pointed out that a chemical speciation model of the buffer solutions, yielding molalities and activities of Tris, TrisH^+^, H^+^ and other species, has a number of potential benefits. These include the extension of the total pH scale to low salinity waters; the ability to calculate the pH of buffers designed for saline waters whose stoichiometry differs from that of seawater; and addressing metrological concerns regarding the traceability of the scale to the International System of Units. Clegg et al.^2^ developed a draft model of Tris buffer in artificial seawater, using the Pitzer equations^5^ for the calculation of activity coefficients. The model is restricted to 25 °C by lack of data, and is of insufficient accuracy. For completion it requires additional thermodynamic parameters based upon measurements of aqueous solutions containing several single solutes (Tris, and cation TrisH^+^ paired with anions SO_4_^2–^, HSO_4_^–^ and Cl^–^), and key mixtures containing Tris or TrisH^+^ (e.g., aqueous HCl–TrisHCl and Tris-NaCl) for a range of temperatures. These data needs are summarized in Table 9 of Clegg et al.^2^ Literature data that can contribute toward the speciation model of buffer solutions include cell potentials of aqueous HCl–TrisHCl mixtures at 25 °C,^6,7^ water activities of aqueous Tris and TrisHCl,^8,9^ cell potentials of Tris buffers in aqueous NaCl,^9^ and solubilities in salt solutions also containing Tris.^10^

This study is the first of a series, for solutions of different compositions, from a collaboration involving the national metrology institutes of Japan (hereinafter NMIJ), Germany, and the USA where all measurements have been carried out. In this work we present results of measurements of electrochemical potentials using Harned cells (which yield activity products of H^+^ and Cl^–^) of aqueous HCl–TrisHCl mixtures over a range of temperatures and ionic strengths. It is intended that these data, in combination with the results of other studies, will enable Pitzer parameters for TrisH^+^–Cl^–^, H^+^–TrisH^+^ and H^+^–TrisH^+^–Cl^–^ interactions to be determined as functions of temperature.

## 2 Experimental Method

In this study the activity products of H^+^ and Cl^–^ ions were determined from measurements of the potential difference of the following electrochemical cell
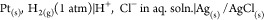
Awhere the aqueous solution contains H^+^, TrisH^+^, and Cl^–^ ions. The potential, *E* (V), of cell A is given by the expression
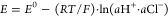
1where *E*^0^ (V) is the standard potential of the cell at the temperature *T* (K) of interest, *R* (8.31446 J mol^–1^ K^–1^) is the gas constant, *F* (96485.332 C mol^–1^) is Faraday’s constant, and prefix *a* denotes activity. The activity product of the H^+^ and Cl^–^ ions can also be written *m*H^+^·*m*Cl^–^·γ_H_·γ_Cl_ or *m*H^+^·*m*Cl^–^·γ_HCl_^2^, where prefix *m* indicates molality, γ_*i*_ is the activity coefficient of individual solute species *i*, and γ_HCl_ is the mean activity coefficient of H^+^ and Cl^–^ ions in the aqueous solution (γ_HCl_ is equal to (γ_H_·γ_Cl_)^0.5^).

**Figure 1:**
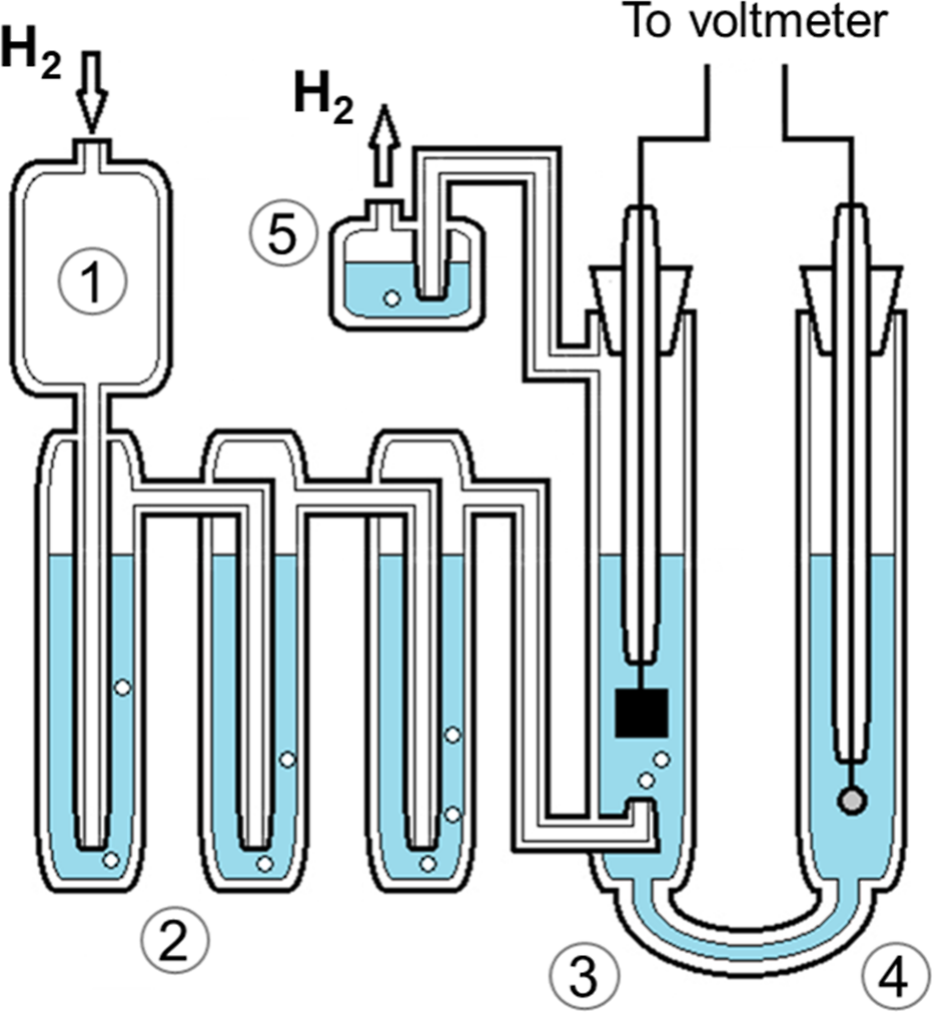
Schematic of a Harned cell. A flow of dry hydrogen enters the damper chamber (1), and then passes through a set of three presaturators (2), and into the half-cell containing the solution being measured and a platinum hydrogen electrode (3). The gas flow exits via a small trap (5). Half-cell (3) is connected, via a glass capillary tube, to half-cell (4) which contains the same solution and a silver–silver chloride electrode. The whole cell is immersed in a water bath for temperature control.

A schematic of the Harned cell (cell A) used at NMIJ is shown in Figure 1. A flow of dry hydrogen gas at a rate of 4 cm^3^ min^–1^ enters the damper chamber (top left of Figure 1) and then passes into a set of three presaturators all of which contain an aqueous solution of the same composition as being measured. The gas flow next passes into the half-cell of the U-shaped measurement compartment containing the platinum hydrogen electrode, and bubbles through the solution. The gas exits the cell via a hydraulic trap designed to prevent any direct contact with the air. This half-cell is connected, with a glass capillary tube of about 1.5 mm internal diameter, to the other half-cell which contains the same solution and the reference silver–silver chloride electrode. For each measurement run a set of six Harned cells is immersed in a water bath, for temperature control, to just above the top of the presaturators.

A total of 18 Harned cells and 12 reference electrodes were used in this study. The preparation of hydrogen and reference electrodes is described by Bates,^11^ and the specific procedures used at NMIJ are summarized in the Supporting Information. The cells were immersed in a Hart Scientific model 7008 constant temperature bath of 42 L capacity (Fluke Corp.), and temperature monitored using a Fluke 1502A thermometer (standard uncertainty ±0.0007 °C). Hydrogen gas was generated using a Parker Balston H2PD-150JA-100 generator (Parker Corp.), and cell potentials were recorded using a model 2182A Keithley Nanovoltmeter (Tektronix). Atmospheric pressure, and hence gas pressure in the cells, was measured using a GE Druck DPI 740 precision barometer (General Electric) (±20 Pa). The setup for Harned cell measurements at NMIJ is described in detail by Ohata.^12^

### 2.1 Solution Compositions and Preparation

The total molal ionic strengths (*I*) of the HCl – TrisHCl aqueous solutions range from 1.0 to 5.0 mol kg^–1^, with H^+^ cation fractions *y*H^+^ [equal to *m*H^+^/(*m*H^+^ + *m*TrisH^+^)] of 0.1, 0.3, and 0.5. In this way the study focuses on those solutions in which the cation TrisH^+^ can be expected to have a generally larger influence on the Cl^–^ activity than will H^+^. It will also affect H^+^ activity. The measurement of a wide range of ionic strengths should enable the unknown Pitzer interaction parameters for this mixture to be determined accurately, because their influence on ln(γ_HCl_) scales with solute species molalities or molality products. The choice of ionic strengths 1.0, 2.0, and 3.0 mol kg^–1^ for some of the measurements enables our results to be compared directly with those of Macaskill and Bates^6^ and Bates and Macaskill^7^ at 25 °C.

The chemicals used in the preparation of the solutions are listed in Table 2. The solid Tris was stored at room temperature, and used directly from the sealed bottles supplied by the manufacturer without additional drying. The concentrated HCl was diluted with ultrapure water to produce stock solutions of lower concentrations, and their exact molalities (3.6455 ± 0.0009, 3.6600 ± 0.0018, 5.7700 ± 0.0028, and 6.3979 ± 0.0009 mol kg^–1^) were determined by coulometric titration.

**Table 2:** Chemicals Used in This Study

chemical	CAS registry #	molar mass	supplier or source	notes
Tris^a^	77-86-1	121.135 g	FUJIFILM WAKO Pure Chemical Corp.	the manufacturer’s certificate stated that the purity was 99.87 mass %, and this value was assumed
HCl	7647-01-0	36.4609 g	Kanto Chemical Co.	ultrapure grade aqueous HCl of 31.4 mass % (diluted with water and then molality determined before use)
H_2_O	7732-18-5	18.0153 g	Milli-Q Ultra Pure Water System (Merck)	resistivity 18.2 MΩ cm at 25 °C

a 2-amino-2-(hydroxymethyl)propane-1,3-diol, C_4_H_11_NO_3_.

For the ∼0.01 mol kg^–1^ HCl required for the determination of the standard potentials of the Harned cells the concentrated HCl was first diluted to make an approximately 0.1 mol kg^–1^ stock solution, and its exact molality was also determined by coulometry. This solution was then diluted gravimetrically to obtain the required 0.01 mol kg^–1^ solutions (0.00996382, 0.0099641, 0.0099638, and 0.0100000 mol kg^–1^ in this work).

All of the studied HCl + TrisHCl solutions were prepared gravimetrically as weights in air of HCl stock solution aliquots, solid Tris, and water. Buoyancy corrections were carried out using equations presented in Dickson et al.,^13^ and assuming a laboratory temperature of 20 °C. Densities of solid Tris of 1.32, 1.328, and about 1.35 g cm^–3^ are listed by various chemical suppliers, and we adopted a value of 1.328 g cm^–3^ for the calculation of the buoyancy correction in this study. Densities of aqueous HCl were taken from Clegg and Wexler,^14^ and those of water from Kell.^15^ All of the HCl + TrisHCl solutions were prepared in duplicate.

### 2.2 Measurements

Cell potentials were measured from 5 to 45 °C for the 1 mol kg^–1^ chloride solutions, and from 5 to 40 °C for the others. Duplicate solutions were measured in all cases, with supplementary determinations of cell potential also made for some solutions at 1, 4, and 5 mol kg^–1^ chloride molality. The standard potentials of the reference electrodes were obtained from measurements of 0.01 mol kg^–1^ HCl solutions. Identifiers for the individual cells used, the chloride or HCl molalities of the solutions, and the dates of measurements are listed in Table 3.

**Table 3:** Cell Identifiers and Dates of Measurements

cells	*m*Cl^–^ (mol kg^–1^)	date	cells	*m*HCl (mol kg^–1^)	date
1–6	1.0	26/07/17	A–F	0.01	21/08/17
7–12	1.0	31/07/17	G–L	0.01	24/08/17
13–18	2.0	02/08/17	M–R	0.01	25/09/17
19–24	2.0	07/08/17	S–X	0.01	23/10/17
25–30	3.0	09/08/17	A1–F1	0.01	14/12/17
31–36	3.0	15/08/17			
37–42	4.0	28/08/17			
43–48	4.0	31/08/17			
49–54	5.0	04/09/17			
55–60	5.0	11/09/17			
1A–6A	1.0	13/09/17			
61–62	1.0	02/10/17			
63–64	4.0	02/10/17			
65–66	5.0	02/10/17			
67–68	4.0	05/10/17			
69–72	5.0	05/10/17			

The Harned cells described above are routinely used for the certification of buffer solutions of ionic strengths up to 0.1 mol kg^–1^, and the measurements in this study presented an additional difficulty. This is because the solubility of AgCl increases greatly in solutions containing high concentrations of chloride ions, for example by a factor of about 50 in ∼5 mol kg^–1^ NaCl_(aq)_ compared to ∼1 mol kg^–1^ NaCl_(aq)_.^16^ In their study of the long-term stability of silver–silver chloride electrodes Maksimov et al.^17^ have described the resulting dissolution and degradation of the electrode, noting that at such high concentrations the electrodeposited layer of silver chloride on the electrode is gradually dissolved due to formation of various aqueous chloro-complexes of the silver cation. In this process the electrode is irreversibly damaged, and it is therefore necessary to measure the solutions in a relatively short space of time.

In dilute buffer solutions the criterion of stability of cell potential is a voltage drift not exceeding 10 μV h^–1^. In this study we observed a similar bias only in the solutions with a chloride molality of 1 mol kg^–1^, and the drift rose to about 50 μV h^–1^ for 2–3 mol kg^–1^ of chloride and was sometimes greater in the more concentrated solutions. In solutions with a chloride molality of 5 mol kg^–1^ it was necessary several times to replace failing electrodes which were identified either by large differences in cell potentials between duplicate measurements, or unacceptable potential drift during a single voltage recording. Discoloration of the platinum hydrogen electrode was observed twice at the end of a series of measurements (resulting in data being discarded) and a possible explanation for this is deposition of silver, from the chloro-complexes mentioned above, onto the electrode (J. F. Waters, pers. comm.). It has been noted by Bates^11^ that the cations of certain metals, such as silver and mercury, can “poison” the electrode by inhibiting its reversibility.

## 3 Treatment of the Data

The measured cell potentials, *E*_meas_, at the ambient H_2_ partial pressure in the cell are corrected to *p*H_2_ equal to 1 atm using the following relationship^11^
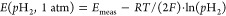
2awhere
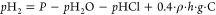
2band *P* (atm) is atmospheric pressure at the time of the measurement, *p*H_2_O (atm) and *p*HCl (atm) are the partial pressures of water and of HCl, respectively, above the solution at the temperature of the measurement. The final term in eq 2b is a further correction in which 0.4 is an empirical factor (due to Hills and Ives^18^ ), ρ (g cm^–3^) is the density of the solution, *h* (mm) is the depth of immersion of the H_2_ electrode, *g* (9.81 m s^–2^) is the gravitational constant, and C (1/101325 atm Pa^–1^) is a conversion factor from Pa to atm. The influences of the different terms in eq 2b on the adjustment to the measured potential are listed in Table 4 for a solution at 5 and 40 °C. The change is more than a factor of 10 greater at the higher temperature because of the much larger influence of the water vapor pressure (*p*H_2_O). The contribution of *p*HCl is negligible at both temperatures.

**Table 4:** Effect of the Terms in the Pressure Correction to the Measured Potentials (eq 2b)^a^

adjustment term contains	5 °C		40 °C	
	mV	cumulative % of total	mV	cumulative % of total
*P*	0.022	20.1	0.131	11.8
*P* – *p*H_2_O	0.122	113.7	1.128	101.6
*P* – *p*H_2_O + JetCorr^b^	0.107	100.0	1.110	100.0
*P* – *p*H_2_O + JetCorr – *p*HCl^c^	0.107	100.0	1.110	100.0

a The examples used here are measurements of a solution containing 1.0 mol kg^–1^Cl^–^, and 0.1 mol kg^–1^ H^+^. The correction term is given by −*RT*/(2*F*)·ln(*X*), where *X* is the quantity listed in the leftmost column.

b The correction for the immersion of the H_2_ bubbler in the cell.

c The correction for *p*HCl is negligible.

The equilibrium partial pressure of water above an aqueous solution is equal to *a*H_2_O·*p*^o^(H_2_O), neglecting the small difference between partial pressure and fugacity, where *a*H_2_O is the water activity of the solution and *p*^o^(H_2_O) (atm) the vapor pressure of pure water at the temperature of the measurement (calculated using the expression of Wagner and Pruss^19^). Values of *a*H_2_O and the H^+^ and Cl^–^ activity coefficients in the solutions were estimated using the Pitzer model, and *p*HCl from the expression *a*H^+^·*a*Cl^–^/*K*_H_ where *K*_H_ (mol^2^ kg^–2^ atm^–1^) is the Henry’s law constant of HCl at the temperature of interest. Densities of the solutions were estimated by assuming additivity of the apparent molar volumes of the electrolytes (HCl and TrisHCl) in the solutions. Further details of the calculation of *a*H_2_O, *p*HCl and ρ are given in the Supporting Information.

### 3.1 Standard Potentials

Standard potentials, *E*^0^, of Cell A at each temperature were obtained from the measurements of 0.01 *m* HCl solutions, adjusted to 1 atm *p*H_2_, together with mean activity coefficients of HCl listed by Bates and Robinson.^20^ The effects of the very small deviations of the solution compositions from exactly 0.01 mol kg^–1^ were compensated for by adjusting the potentials *E* according to the equation
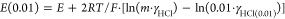
3where *E*(0.01) is the estimated potential of exactly 0.01 *m* HCl, *E* is that of the solution containing HCl of molality *m* (where *m* is very close to 0.01), and mean activity coefficients γ_HCl_ and γ_HCl(0.01)_ are values calculated for pure aqueous HCl of molality *m* and 0.01, respectively, using the equations of Holmes et al.^21^ (with the first set of parameters in their Table 3). Values of the standard potentials, *E*^0^, at each temperature were obtained using *E*(0.01) and HCl mean activity coefficients from Bates and Robinson^20^ in eq 1. Information concerning the cells used to determine the standard potentials at each temperature, and the values of *E*^0^ (with uncertainties) determined in this study, can be found in the Supporting Information.

It is common in Harned cell studies for the potentials of the measurement solutions to be adjusted to a common set of standard potentials, those of Bates and Bower,^22^ for consistency and ease of comparability. We have done this here. The potentials, *E*, of the measurement solutions for *p*H_2_ equal to 1 atm were adjusted using the following expression
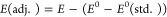
4where *E* (adj.) are the potentials adjusted to be consistent with the standard potentials of Bates and Bower,^22^*E* and standard potentials *E*^0^ are the values obtained in this study, and *E*^0^(std.) are from eq 4 of Bates and Bower and listed in their Table 1.

### 3.2 Uncertainties

The contributions to the overall uncertainty of a measured potential, after adjustment to *p*H_2_ equal to 1 atm, are as follows: 96 to 99% is from the voltage measurements themselves; the determination of barometric pressure contributes about 1–4%; and other elements (temperature, solution water activities and densities) less than about 0.01%. The uncertainty of the measured potential (that of the voltmeter was ignored as negligible) was calculated as a combined value of cell potential drift at the experimental temperature and the standard deviation SD of two (or four or six, if available) duplicate measurements:
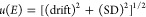
5where the potential drift over a period of 1 h experienced in the measurements is equal to 10 μV at the temperatures 5, 10, 15, and 20 °C; 20 μV at the temperatures 25, 30, and 35 °C; and 40 μV at the temperatures 40 and 45 °C. We note that the drift values were slightly higher for the solutions used in the determination of *E*^0^, because the dilute aqueous HCl seems to be more sensitive to the effects of evaporation.

In most cases, duplicate measurements agreed very well—to within 10 μV—so that the determined *u*(*E*) is close to the drift value. However, for solutions for which there was a greater number of determinations the calculated value of *u*(*E*) typically rises to 80–150 μV. This is because we made the extra replicate measurements for the solution compositions with obviously doubtful original results, i.e. with a high discrepancy between duplicate cells (perhaps due to the effect of accumulated electrode degradation).

## 4 Results

Measured cell potentials, corrected to *p*H_2_ equal to 1 atm and adjusted to be consistent with the standard potentials of Bates and Bower^22^ are listed in Table 5 together with mean activity coefficients of HCl, γ_HCl_, calculated using eq 1. In Table S8 of the Supporting Information the original measured potentials are listed, together with other information needed in eq 2, and also the estimated uncertainties in γ_HCl_. A total of four results were removed as erroneous (and are not listed), due to very large calculated uncertainties and/or deviations from other measurements.

**Table 5:** Harned Cell Results including Calculated Mean Activity Coefficients of HCl^a^

*t*^b^ (°C)	*m*Cl^–^ (mol kg^–1^)	*y*H^+^	*m*HCl^c^ (mol kg^–1^)	*m*TrisHCl (mol kg^–1^)^c^	*E*(adj.) (V)^d^	*u*(*E*) (mV)	γ_HCl_		*t*^b^ (°C)	*m*Cl^–^ (mol kg^–1^)	*y*H^+^	*m*HCl^c^ (mol kg^–1^)	*m*TrisHCl (mol kg^–1^)^c^	*E*(adj.) (V)^d^	*u*(*E*) (mV)	γ_HCl_
5	1.0	0.10	0.10001	0.90027	0.30503	0.144	0.7197		25	3.0	0.50	1.50131	1.50127	0.18367	0.080	1.0007
5	1.0	0.10	0.10001	0.90025	0.30468	0.144	0.7250		25	3.0	0.50	1.50121	1.50126	0.18357	0.080	1.0029
5	1.0	0.30	0.30010	0.70020	0.27680	0.010	0.7486		30	3.0	0.10	0.30016	2.70218	0.23205	0.020	0.8221
5	1.0	0.30	0.30011	0.70018	0.27680	0.010	0.7486		30	3.0	0.10	0.30032	2.70213	0.23205	0.020	0.8220
5	1.0	0.50	0.50016	0.50011	0.26308	0.010	0.7721		30	3.0	0.30	0.90074	2.10170	0.19873	0.020	0.8981
5	1.0	0.50	0.50013	0.50013	0.26308	0.010	0.7722		30	3.0	0.30	0.90078	2.10181	0.19873	0.020	0.8980
10	1.0	0.10	0.10001	0.90027	0.30391	0.141	0.7154		30	3.0	0.50	1.50117	1.50114	0.18047	0.085	0.9866
10	1.0	0.10	0.10001	0.90025	0.30357	0.141	0.7205		30	3.0	0.50	1.50114	1.50117	0.18035	0.085	0.9889
10	1.0	0.30	0.30010	0.70020	0.27521	0.010	0.7438		40	3.0	0.10	0.30016	2.70218	0.22707	0.040	0.7978
10	1.0	0.30	0.30011	0.70018	0.27521	0.010	0.7437		40	3.0	0.10	0.30032	2.70213	0.22707	0.040	0.7976
10	1.0	0.50	0.50016	0.50011	0.26126	0.010	0.7668		40	3.0	0.30	0.90074	2.10170	0.19278	0.040	0.8693
10	1.0	0.50	0.50013	0.50013	0.26125	0.010	0.7668		40	3.0	0.30	0.90078	2.10181	0.19278	0.040	0.8693
15	1.0	0.10	0.10001	0.90027	0.30269	0.118	0.7106		40	3.0	0.50	1.50117	1.50114	0.17399	0.041	0.9539
15	1.0	0.10	0.10001	0.90025	0.30235	0.118	0.7155		40	3.0	0.50	1.50114	1.50117	0.17398	0.041	0.9541
15	1.0	0.30	0.30010	0.70020	0.27349	0.061	0.7386		5	4.0	0.10	0.40025	3.60202	0.22388	0.012	0.9775
15	1.0	0.30	0.30011	0.70018	0.27349	0.061	0.7386		5	4.0	0.10	0.40021	3.60218	0.22387	0.012	0.9777
15	1.0	0.50	0.50016	0.50011	0.25932	0.060	0.7609		5	4.0	0.30	1.20070	2.80167	0.19137	0.010	1.1119
15	1.0	0.50	0.50013	0.50013	0.25932	0.060	0.7610		5	4.0	0.30	1.20067	2.80165	0.19137	0.010	1.1119
15	1.0	0.10	0.10004	0.90020	0.30245	0.118	0.7140		5	4.0	0.50	2.00127	2.00128	0.17245	0.014	1.2780
15	1.0	0.10	0.10000	0.90026	0.30245	0.118	0.7141		5	4.0	0.50	2.00122	2.00118	0.17246	0.014	1.2778
15	1.0	0.30	0.30006	0.70020	0.27359	0.061	0.7371		10	4.0	0.10	0.40022	3.60222	0.22166	0.011	0.9647
15	1.0	0.30	0.30007	0.70018	0.27360	0.061	0.7370		10	4.0	0.10	0.40017	3.60199	0.22165	0.011	0.9649
15	1.0	0.50	0.50007	0.50012	0.25941	0.060	0.7598		10	4.0	0.30	1.20075	2.80174	0.18861	0.070	1.0963
15	1.0	0.50	0.50015	0.50014	0.25926	0.060	0.7619		10	4.0	0.30	1.20071	2.80166	0.18876	0.070	1.0929
20	1.0	0.10	0.10004	0.90021	0.30104	0.137	0.7097		10	4.0	0.50	2.00123	2.00116	0.16954	0.017	1.2552
20	1.0	0.10	0.10004	0.90025	0.30092	0.137	0.7114		10	4.0	0.50	2.00122	2.00120	0.16955	0.017	1.2551
20	1.0	0.30	0.30009	0.70021	0.27166	0.010	0.7331		15	4.0	0.10	0.40025	3.60202	0.21947	0.011	0.9488
20	1.0	0.30	0.30008	0.70017	0.27166	0.010	0.7331		15	4.0	0.10	0.40021	3.60218	0.21946	0.011	0.9490
20	1.0	0.50	0.50016	0.50011	0.25729	0.011	0.7546		15	4.0	0.30	1.20070	2.80167	0.18598	0.011	1.0753
20	1.0	0.50	0.50017	0.50012	0.25728	0.011	0.7547		15	4.0	0.30	1.20067	2.80165	0.18597	0.011	1.0754
25	1.0	0.10	0.10004	0.90020	0.29958	0.077	0.7040		15	4.0	0.50	2.00127	2.00128	0.16659	0.011	1.2306
25	1.0	0.10	0.10000	0.90026	0.29958	0.077	0.7041		15	4.0	0.50	2.00122	2.00118	0.16659	0.011	1.2306
25	1.0	0.30	0.30006	0.70020	0.26976	0.020	0.7262		20	4.0	0.10	0.40025	3.60202	0.21713	0.011	0.9337
25	1.0	0.30	0.30007	0.70018	0.26976	0.020	0.7262		20	4.0	0.10	0.40021	3.60218	0.21713	0.011	0.9338
25	1.0	0.50	0.50007	0.50012	0.25510	0.099	0.7483		20	4.0	0.30	1.20070	2.80167	0.18311	0.011	1.0570
25	1.0	0.50	0.50015	0.50014	0.25496	0.099	0.7502		20	4.0	0.30	1.20067	2.80165	0.18311	0.011	1.0571
30	1.0	0.10	0.10004	0.90021	0.29793	0.101	0.6991		20	4.0	0.50	2.00127	2.00128	0.16351	0.011	1.2068
30	1.0	0.10	0.10004	0.90025	0.29780	0.101	0.7009		20	4.0	0.50	2.00122	2.00118	0.16351	0.011	1.2070
30	1.0	0.30	0.30009	0.70021	0.26756	0.020	0.7219		25	4.0	0.10	0.40025	3.60202	0.21467	0.021	0.9183
30	1.0	0.30	0.30008	0.70017	0.26756	0.020	0.7219		25	4.0	0.10	0.40021	3.60218	0.21466	0.021	0.9185
30	1.0	0.50	0.50016	0.50011	0.25269	0.020	0.7433		25	4.0	0.30	1.20070	2.80167	0.18016	0.020	1.0379
30	1.0	0.50	0.50017	0.50012	0.25269	0.020	0.7433		25	4.0	0.30	1.20067	2.80165	0.18016	0.020	1.0379
35	1.0	0.10	0.10004	0.90020	0.29625	0.020	0.6932		25	4.0	0.50	2.00127	2.00128	0.16039	0.054	1.1811
35	1.0	0.10	0.10000	0.90026	0.29625	0.020	0.6932		25	4.0	0.50	2.00122	2.00118	0.16027	0.054	1.1839
35	1.0	0.30	0.30006	0.70020	0.26548	0.020	0.7144		30	4.0	0.10	0.40022	3.60222	0.21205	0.021	0.9042
35	1.0	0.30	0.30007	0.70018	0.26547	0.020	0.7145		30	4.0	0.10	0.40017	3.60199	0.21205	0.021	0.9044
35	1.0	0.50	0.50007	0.50012	0.25036	0.022	0.7356		30	4.0	0.30	1.20075	2.80174	0.17707	0.039	1.0198
35	1.0	0.50	0.50015	0.50014	0.25022	0.022	0.7376		30	4.0	0.30	1.20071	2.80166	0.17707	0.039	1.0197
40	1.0	0.10	0.10004	0.90021	0.29440	0.150	0.6876		30	4.0	0.50	2.00122	2.00120	0.15697	0.122	1.1605
40	1.0	0.10	0.10004	0.90025	0.29425	0.150	0.6895		40	4.0	0.10	0.40022	3.60222	0.20669	0.040	0.8729
40	1.0	0.30	0.30009	0.70021	0.26308	0.040	0.7093		40	4.0	0.10	0.40017	3.60199	0.20668	0.040	0.8731
40	1.0	0.30	0.30008	0.70017	0.26309	0.040	0.7092		40	4.0	0.30	1.20075	2.80174	0.17074	0.075	0.9810
40	1.0	0.50	0.50016	0.50011	0.24769	0.040	0.7308		40	4.0	0.30	1.20071	2.80166	0.17088	0.075	0.9786
40	1.0	0.50	0.50017	0.50012	0.24769	0.040	0.7307		40	4.0	0.50	2.00122	2.00120	0.15013	0.075	1.1132
45	1.0	0.10	0.10004	0.90020	0.29248	0.040	0.6815		5	4.0	0.50	2.00125	2.00127	0.17247	0.014	1.2776
45	1.0	0.10	0.10000	0.90026	0.29248	0.040	0.6816		5	4.0	0.50	2.00143	2.00143	0.17247	0.014	1.2774
45	1.0	0.30	0.30006	0.70020	0.26074	0.083	0.7020		10	4.0	0.50	2.00125	2.00127	0.16957	0.017	1.2545
45	1.0	0.30	0.30007	0.70018	0.26064	0.083	0.7033		10	4.0	0.50	2.00143	2.00143	0.16957	0.017	1.2544
45	1.0	0.50	0.50007	0.50012	0.24520	0.123	0.7220		10	4.0	0.30	1.20079	2.80189	0.18873	0.070	1.0935
45	1.0	0.50	0.50015	0.50014	0.24503	0.123	0.7241		10	4.0	0.30	1.20086	2.80187	0.18874	0.070	1.0933
5	1.0	0.10	0.10003	0.90026	0.30475	0.144	0.7239		15	4.0	0.50	2.00125	2.00127	0.16659	0.011	1.2306
5	1.0	0.10	0.10005	0.90022	0.30474	0.144	0.7239		15	4.0	0.50	2.00143	2.00143	0.16659	0.011	1.2304
10	1.0	0.10	0.10003	0.90026	0.30363	0.141	0.7195		20	4.0	0.50	2.00125	2.00127	0.16350	0.011	1.2071
10	1.0	0.10	0.10005	0.90022	0.30363	0.141	0.7195		20	4.0	0.50	2.00143	2.00143	0.16350	0.011	1.2069
15	1.0	0.10	0.10003	0.90026	0.30243	0.118	0.7143		25	4.0	0.50	2.00125	2.00127	0.16033	0.054	1.1824
15	1.0	0.10	0.10005	0.90022	0.30242	0.118	0.7143		25	4.0	0.50	2.00143	2.00143	0.16033	0.054	1.1822
20	1.0	0.10	0.10003	0.90026	0.30108	0.137	0.7093		30	4.0	0.50	2.00125	2.00127	0.15703	0.122	1.1591
20	1.0	0.10	0.10005	0.90022	0.30107	0.137	0.7093		30	4.0	0.50	2.00143	2.00143	0.15703	0.122	1.1591
25	1.0	0.10	0.10003	0.90026	0.29958	0.077	0.7040		30	4.0	0.30	1.20079	2.80189	0.17713	0.039	1.0186
25	1.0	0.10	0.10005	0.90022	0.29957	0.077	0.7040		30	4.0	0.30	1.20086	2.80187	0.17713	0.039	1.0184
30	1.0	0.10	0.10003	0.90026	0.29795	0.101	0.6990		40	4.0	0.50	2.00125	2.00127	0.15026	0.075	1.1105
30	1.0	0.10	0.10005	0.90022	0.29794	0.101	0.6990		40	4.0	0.50	2.00143	2.00143	0.15026	0.075	1.1106
40	1.0	0.10	0.10003	0.90026	0.29443	0.150	0.6872		40	4.0	0.30	1.20079	2.80189	0.17086	0.075	0.9788
40	1.0	0.10	0.10005	0.90022	0.29442	0.150	0.6873		40	4.0	0.30	1.20086	2.80187	0.17087	0.075	0.9787
5	2.0	0.10	0.20008	1.80102	0.26738	0.010	0.7890		5	5.0	0.10	0.50031	4.50318	0.20843	0.147	1.0792
5	2.0	0.10	0.20009	1.80101	0.26738	0.010	0.7890		5	5.0	0.10	0.50037	4.50334	0.20813	0.147	1.0859
5	2.0	0.30	0.60024	1.40047	0.23804	0.010	0.8402		5	5.0	0.30	1.50108	3.50256	0.17404	0.011	1.2767
5	2.0	0.30	0.60033	1.40081	0.23804	0.010	0.8400		5	5.0	0.30	1.50112	3.50270	0.17405	0.011	1.2765
5	2.0	0.50	1.00058	1.00060	0.22276	0.010	0.8950		5	5.0	0.50	2.50185	2.50192	0.15322	0.012	1.5269
5	2.0	0.50	1.00048	1.00052	0.22276	0.010	0.8951		5	5.0	0.50	2.50197	2.50191	0.15321	0.012	1.5271
10	2.0	0.10	0.20011	1.80096	0.26595	0.010	0.7785		10	5.0	0.10	0.50040	4.50337	0.20588	0.095	1.0662
10	2.0	0.10	0.20011	1.80100	0.26595	0.010	0.7786		10	5.0	0.10	0.50036	4.50341	0.20587	0.095	1.0663
10	2.0	0.30	0.60037	1.40075	0.23589	0.011	0.8321		10	5.0	0.30	1.50115	3.50282	0.17120	0.055	1.2529
10	2.0	0.30	0.60034	1.40073	0.23590	0.011	0.8321		10	5.0	0.30	1.50114	3.50274	0.17133	0.055	1.2496
10	2.0	0.50	1.00056	1.00057	0.22042	0.011	0.8850		10	5.0	0.50	2.50189	2.50195	0.15018	0.159	1.4928
10	2.0	0.50	1.00057	1.00052	0.22042	0.011	0.8850		10	5.0	0.50	2.50184	2.50184	0.14987	0.159	1.5025
15	2.0	0.10	0.20008	1.80102	0.26405	0.010	0.7734		15	5.0	0.10	0.50031	4.50318	0.20367	0.106	1.0433
15	2.0	0.10	0.20009	1.80101	0.26405	0.010	0.7734		15	5.0	0.10	0.50037	4.50334	0.20342	0.106	1.0485
15	2.0	0.30	0.60024	1.40047	0.23370	0.010	0.8228		15	5.0	0.30	1.50108	3.50256	0.16827	0.010	1.2286
15	2.0	0.30	0.60033	1.40081	0.23370	0.010	0.8226		15	5.0	0.30	1.50112	3.50270	0.16827	0.010	1.2285
15	2.0	0.50	1.00058	1.00060	0.21797	0.010	0.8747		15	5.0	0.50	2.50185	2.50192	0.14702	0.096	1.4597
15	2.0	0.50	1.00048	1.00052	0.21797	0.010	0.8748		15	5.0	0.50	2.50197	2.50191	0.14689	0.096	1.4635
20	2.0	0.10	0.20008	1.80102	0.26219	0.011	0.7656		20	5.0	0.10	0.50031	4.50318	0.20105	0.096	1.0269
20	2.0	0.10	0.20009	1.80101	0.26219	0.011	0.7656		20	5.0	0.10	0.50037	4.50334	0.20085	0.096	1.0308
20	2.0	0.30	0.60024	1.40047	0.23135	0.011	0.8139		20	5.0	0.30	1.50108	3.50256	0.16523	0.010	1.2045
20	2.0	0.30	0.60033	1.40081	0.23135	0.011	0.8137		20	5.0	0.30	1.50112	3.50270	0.16523	0.010	1.2045
20	2.0	0.50	1.00058	1.00060	0.21538	0.011	0.8646		20	5.0	0.50	2.50185	2.50192	0.14375	0.094	1.4273
20	2.0	0.50	1.00048	1.00052	0.21538	0.011	0.8647		20	5.0	0.50	2.50197	2.50191	0.14362	0.094	1.4310
25	2.0	0.10	0.20008	1.80102	0.26021	0.020	0.7572		25	5.0	0.10	0.50031	4.50318	0.19832	0.059	1.0099
25	2.0	0.10	0.20009	1.80101	0.26021	0.020	0.7572		25	5.0	0.10	0.50037	4.50334	0.19831	0.059	1.0100
25	2.0	0.30	0.60024	1.40047	0.22888	0.020	0.8045		25	5.0	0.30	1.50108	3.50256	0.16213	0.020	1.1791
25	2.0	0.30	0.60033	1.40081	0.22888	0.020	0.8043		25	5.0	0.30	1.50112	3.50270	0.16212	0.020	1.1792
25	2.0	0.50	1.00058	1.00060	0.21271	0.021	0.8533		25	5.0	0.50	2.50185	2.50192	0.14042	0.107	1.3935
25	2.0	0.50	1.00048	1.00052	0.21271	0.021	0.8535		25	5.0	0.50	2.50197	2.50191	0.14027	0.107	1.3975
30	2.0	0.10	0.20011	1.80096	0.25832	0.021	0.7459		30	5.0	0.10	0.50040	4.50337	0.19557	0.052	0.9914
30	2.0	0.10	0.20011	1.80100	0.25832	0.021	0.7460		30	5.0	0.10	0.50036	4.50341	0.19557	0.052	0.9916
30	2.0	0.30	0.60037	1.40075	0.22630	0.020	0.7948		30	5.0	0.30	1.50115	3.50282	0.15896	0.024	1.1536
30	2.0	0.30	0.60034	1.40073	0.22631	0.020	0.7948		30	5.0	0.30	1.50114	3.50274	0.15896	0.024	1.1535
30	2.0	0.50	1.00056	1.00057	0.20987	0.020	0.8432		30	5.0	0.50	2.50189	2.50195	0.13690	0.110	1.3630
30	2.0	0.50	1.00057	1.00052	0.20987	0.020	0.8433		30	5.0	0.50	2.50184	2.50184	0.13668	0.110	1.3687
40	2.0	0.10	0.20011	1.80096	0.25388	0.081	0.7283		40	5.0	0.10	0.50040	4.50337	0.18991	0.280	0.9527
40	2.0	0.10	0.20011	1.80100	0.25377	0.081	0.7297		40	5.0	0.10	0.50036	4.50341	0.18992	0.280	0.9527
40	2.0	0.30	0.60037	1.40075	0.22086	0.041	0.7753		40	5.0	0.30	1.50115	3.50282	0.15233	0.082	1.1037
40	2.0	0.30	0.60034	1.40073	0.22086	0.041	0.7752		40	5.0	0.30	1.50114	3.50274	0.15244	0.082	1.1014
40	2.0	0.50	1.00056	1.00057	0.20397	0.040	0.8211		40	5.0	0.50	2.50189	2.50195	0.12927	0.243	1.3107
40	2.0	0.50	1.00057	1.00052	0.20397	0.040	0.8212		40	5.0	0.50	2.50184	2.50184	0.12959	0.243	1.3031
5	3.0	0.10	0.30024	2.70224	0.24280	0.010	0.8781		5	5.0	0.10	0.50044	4.50392	0.20842	0.147	1.0792
5	3.0	0.10	0.30023	2.70228	0.24280	0.010	0.8781		5	5.0	0.10	0.50048	4.50376	0.20842	0.147	1.0793
5	3.0	0.30	0.90080	2.10180	0.21194	0.010	0.9650		10	5.0	0.10	0.50044	4.50392	0.20604	0.095	1.0626
5	3.0	0.30	0.90078	2.10176	0.21194	0.010	0.9650		10	5.0	0.10	0.50048	4.50376	0.20603	0.095	1.0626
5	3.0	0.50	1.50131	1.50127	0.19485	0.010	1.0675		10	5.0	0.30	1.50140	3.50306	0.17125	0.055	1.2514
5	3.0	0.50	1.50121	1.50126	0.19486	0.010	1.0676		10	5.0	0.30	1.50127	3.50292	0.17125	0.055	1.2514
10	3.0	0.10	0.30016	2.70218	0.24082	0.010	0.8685		10	5.0	0.50	2.50215	2.50210	0.14983	0.159	1.5034
10	3.0	0.10	0.30032	2.70213	0.24082	0.010	0.8682		10	5.0	0.50	2.50217	2.50212	0.15000	0.159	1.4981
10	3.0	0.30	0.90074	2.10170	0.20948	0.087	0.9528		15	5.0	0.10	0.50044	4.50392	0.20359	0.106	1.0447
10	3.0	0.30	0.90078	2.10181	0.20960	0.087	0.9504		15	5.0	0.10	0.50048	4.50376	0.20359	0.106	1.0447
10	3.0	0.50	1.50117	1.50114	0.19218	0.090	1.0523		20	5.0	0.10	0.50044	4.50392	0.20104	0.096	1.0268
10	3.0	0.50	1.50114	1.50117	0.19205	0.090	1.0550		20	5.0	0.10	0.50048	4.50376	0.20104	0.096	1.0268
15	3.0	0.10	0.30024	2.70224	0.23886	0.010	0.8560		25	5.0	0.10	0.50044	4.50392	0.19841	0.059	1.0079
15	3.0	0.10	0.30023	2.70228	0.23886	0.010	0.8560		25	5.0	0.10	0.50048	4.50376	0.19841	0.059	1.0078
15	3.0	0.30	0.90080	2.10180	0.20698	0.010	0.9390		30	5.0	0.10	0.50044	4.50392	0.19565	0.052	0.9898
15	3.0	0.30	0.90078	2.10176	0.20698	0.010	0.9390		30	5.0	0.10	0.50048	4.50376	0.19565	0.052	0.9897
15	3.0	0.50	1.50131	1.50127	0.18948	0.011	1.0346		30	5.0	0.30	1.50140	3.50306	0.15898	0.024	1.1529
15	3.0	0.50	1.50121	1.50126	0.18948	0.011	1.0347		30	5.0	0.30	1.50127	3.50292	0.15898	0.024	1.1529
20	3.0	0.10	0.30024	2.70224	0.23671	0.011	0.8448		30	5.0	0.50	2.50217	2.50212	0.13682	0.110	1.3650
20	3.0	0.10	0.30023	2.70228	0.23671	0.011	0.8448		40	5.0	0.10	0.50044	4.50392	0.18946	0.280	0.9608
20	3.0	0.30	0.90080	2.10180	0.20433	0.010	0.9258		40	5.0	0.10	0.50048	4.50376	0.19011	0.280	0.9492
20	3.0	0.30	0.90078	2.10176	0.20433	0.010	0.9258		40	5.0	0.30	1.50140	3.50306	0.15232	0.082	1.1038
20	3.0	0.50	1.50131	1.50127	0.18664	0.012	1.0178		40	5.0	0.30	1.50127	3.50292	0.15245	0.082	1.1011
20	3.0	0.50	1.50121	1.50126	0.18663	0.012	1.0181		40	5.0	0.50	2.50217	2.50212	0.12974	0.243	1.2992
25	3.0	0.10	0.30024	2.70224	0.23445	0.020	0.8331									
25	3.0	0.10	0.30023	2.70228	0.23445	0.020	0.8331									
25	3.0	0.30	0.90080	2.10180	0.20158	0.020	0.9118									
25	3.0	0.30	0.90078	2.10176	0.20158	0.020	0.9118									

a Columns *m*Cl^–^ and *y*H^+^ contain rounded values, and exact values can be calculated from the listed *m*HCl and *m*TrisHCl.

b Measurement temperatures are equal to 5.000, 10.000 °C, etc. within the expanded uncertainty of the thermometer (±0.007 °C, *k* = 2 from the calibration certificate), and are presented here as integer values.

c Molalities *m*HCl and *m*TrisHCl below 1 mol kg^–1^ are presented with five significant digits, rather than six.

d The potentials for cells 61 and 62, containing 1.0 mol kg^–1^*m*Cl^–^ and with *y*H^+^ equal to 0.1, were adjusted at all temperatures by adding a fixed Δ*E* of −0.0001289 V to the original value of *E*(adj.). This Δ*E* is the value required to bring the potentials of cells 61 and 62 into agreement with other data for the same solution compositions at 25 °C. The cell numbers for all data can be found in the more complete tabulation of results in the Supporting Information (Table S8).

**Figure 2:**
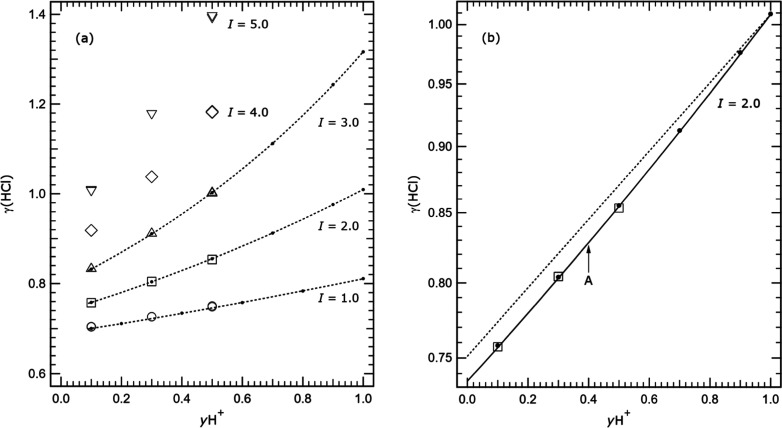
Measured mean activity coefficients γ_HCl_ (labeled γ(HCl) for clarity) at 25 °C plotted against the H^+^ cation fraction *y*H^+^ [*m*H^+^/(*m*H^+^ + *m*TrisH^+^)]. (a) Results at ionic strengths (*I*) of 1.0 to 5.0 mol kg^–1^, as indicated. Symbols: solid dot – data of Macaskill and Bates^6^ (*I* = 1.0 mol kg^–1^), and Bates and Macaskill^7^ (*I* = 2.0 and 3.0 mol kg^–1^); other symbols – this study, at the indicated ionic strengths. Dotted line – fitted to the data from the two studies of Bates and co-workers in order to indicate the variation of γ_HCl_ with *y*H^+^. (b) Results at ionic strength 2.0 mol kg^–1^ only (*y* axis tick marks are spaced logarithmically). Symbols: solid dot – data of Bates and Macaskill;^7^ open square – this study. Solid line ‘A’ – calculated using the model of Clegg et al.^2^ including Pitzer mixture parameters θ_H,TrisH_ and ψ_H,TrisH,Cl_ from Bates and Macaskill;^7^ dotted line – calculated with the same model but without the two mixture parameters.

Macaskill and Bates^6^ and, later, Bates and Macaskill^7^ have used Harned cells to measure cell potentials in the same solutions at 25 °C only and for a series of fixed ionic strengths (*I*) from 0.1 to 3.0 mol kg^–1^. Our results are compared with theirs in Figure 2a at the common ionic strengths, and show good agreement. Bates and Macaskill^7^ have analyzed their results in terms of Harned’s rule, which implies that ln(γ_HCl_) should be a linear function of *m*TrisHCl (and similarly for fraction *y*H^+^) and they found that an additional term in *m*TrisHCl^2^ is needed to fit the data at ionic strength 1.0 mol kg^–1^ and above (their Table V). Our results in Figure 2, especially those at the higher ionic strengths, are consistent with this.

Bates and Macaskill^7^ have also analyzed their results using the Pitzer model, and obtained mixture parameters θ_H,TrisH_ equal to 0.0045 and ψ_H,TrisH,Cl_ equal to −0.0152 (their Table VI). In Figure 2b we compare measured mean activity coefficients γ_HCl_ from this study, and from Bates and Macaskill,^7^ with two sets of calculated values at an ionic strength of 2.0 mol kg^–1^. The *y* axis tickmarks on this plot are spaced logarithmically, so that straight line relationships correspond to Harned’s rule. The solid line was calculated using the Pitzer model of Clegg et al.^2^ (which contains interaction parameters for H^+^-Cl^–^ and TrisH^+^-Cl^–^) and also the ternary mixture parameters θ_H,TrisH_ and ψ_H,TrisH,Cl_ from Table VI of Bates and Macaskill.^7^ There is excellent agreement: the mean deviation of the activity coefficients γ_HCl_ of Macaskill and Bates^6^ from the model-calculated values is 0.11%, and that of the data of this study 0.032%. The results confirm the finding of Bates and Macaskill^7^ that the solute activity coefficients in these solutions can be accurately represented by the Pitzer model as long as the mixture parameters are included. The dotted line in Figure 2b shows the values calculated using the Pitzer model *without* these parameters.

We have not applied the Pitzer model to the results for temperatures other than 25 °C because the values of the parameters for TrisH^+^-Cl^–^ interactions (β_TrisH,Cl_^(0)^, β_TrisH,Cl_^(1)^, *C*_TrisH,Cl_^(0)^, and possibly *C*_TrisH,Cl_^(1)^) are not yet clearly established. They have been estimated by Tishchenko^9^ from 0 to 40 °C from measurements using two electrochemical cells, but our analyses^10^ suggest that at least some of the data may be in error. The TrisH^+^-Cl^–^ parameters can in principle be determined from the results in the present study, but it is not possible to completely distinguish them from the two mixture parameters. Consideration of the Pitzer model expression for ln(γ_H_·γ_Cl_), for example by using equations AI2 and AI3 of Clegg et al.,^23^ or eqs 63 and 64 of Pitzer,^5^ yields the contributions +2*m*TrisH^+^·(β_TrisH,Cl_^(0)^ + θ_H,TrisH_), and + *m*TrisH^+^·(4*m*Cl^–^·*C*_TrisH,Cl_^(0)^ + [*m*H^+^ + *m*Cl^–^]·ψ_H,TrisH,Cl_). We note that the contributions of TrisH^+^-Cl^–^ parameters β_TrisH,Cl_^(1)^ and *C*_TrisH,Cl_^(1)^ are more complex functions of solution composition (and different from θ_H,TrisH_ or ψ_H,TrisH,Cl_). For these reasons a Pitzer model application to obtain the parameters for both TrisH^+^-Cl^–^ and mixture parameters will be carried out in a future work and based upon several different data sets for solutions containing the ions TrisH^+^ and Cl^–^, including some now in preparation.

**Figure 3:**
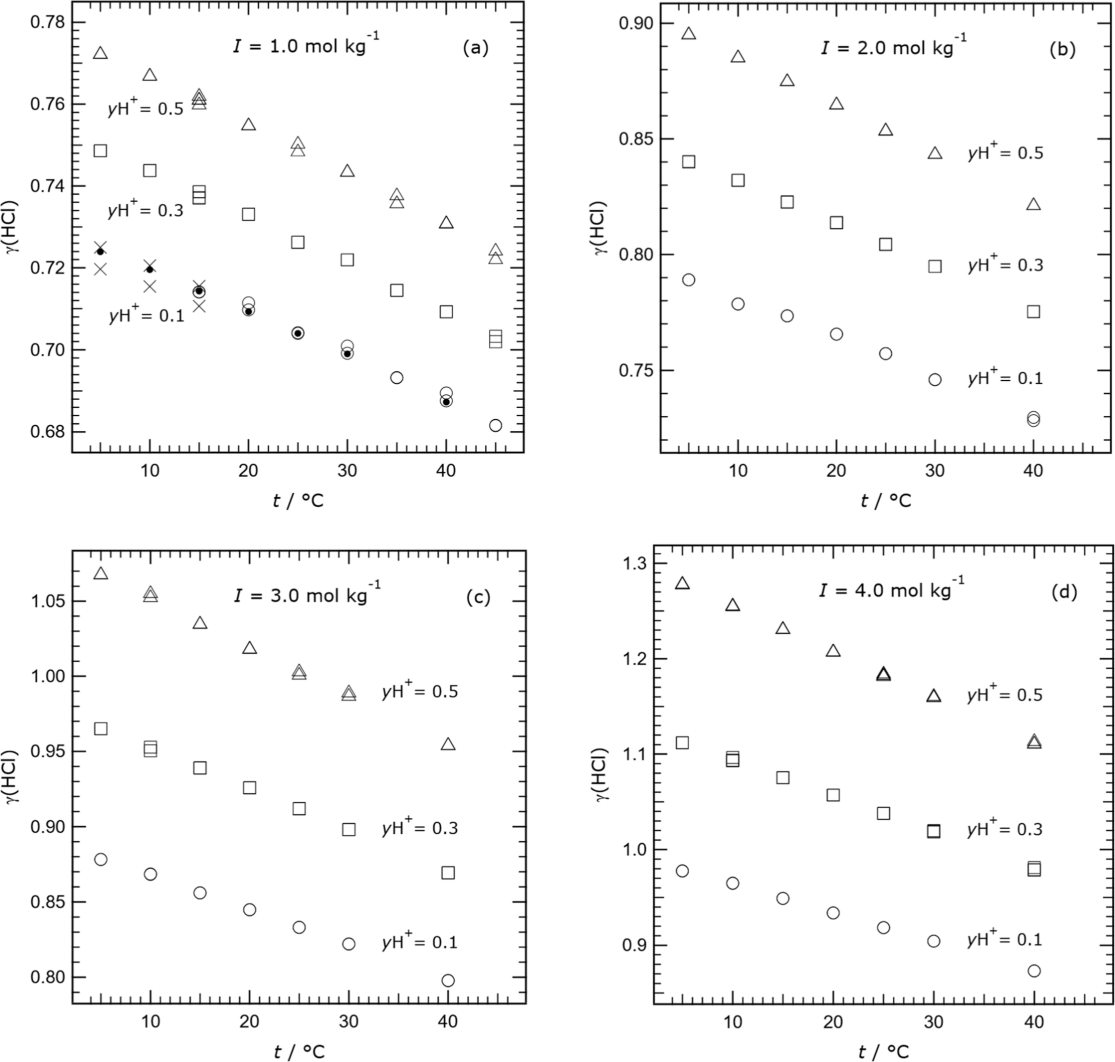
Measured mean activity coefficients γ_HCl_ (labeled γ(HCl) for clarity), obtained in this study for different ionic strengths (*I*) and H^+^ cation fractions *y*H^+^ (*m*H^+^/(*m*H^+^ + *m*TrisH^+^)), plotted against temperature (*t*). Symbols: solid dot, open circle, and cross *– y*H^+^ equal to 0.1; square *– y*H^+^ equal to 0.3; triangle *– y*H^+^ equal to 0.5. (a–d) Results for four different ionic strengths as indicated. In plot (a) the solid dots indicate values for measurements for cells 61 and 62 that have been adjusted (see text), and the crosses are results for cells 1A and 2A. Results for *I* equal to 5.0 mol kg^–1^ are similar and are not shown.

**Figure 4:**
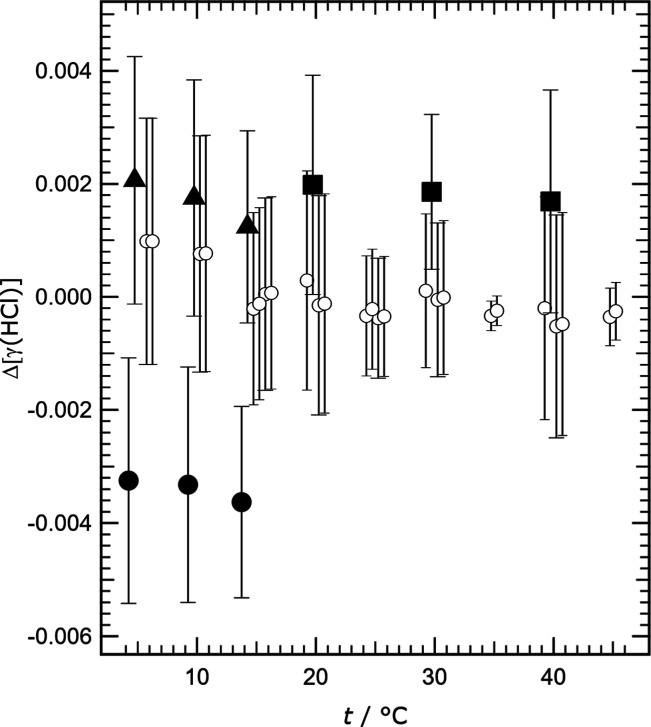
Deviations of measured mean activity coefficients of HCl from an arbitrary fitted line (Δ[γ(HCl)]) plotted against temperature (*t*), for *m*Cl^–^ equal to 1.0 mol kg^–1^ and *y*H^+^ equal to 0.1. Symbols: open circles – from cells 1, 2, 7, 61, and 62; dots – cell 1A; solid triangle – cell 2A; solid square – cell 8. The uncertainties shown on the plot were taken from Table S8 of the Supporting Information. Results for each cell, at each temperature, are offset horizontally so that the error bars are distinguishable.

In Figure 3 we show activity coefficients from Table 5 for ionic strengths 1.0 to 4.0 mol kg^–1^ as a function of temperature. The variation in γ_HCl_ with temperature is very close to linear in all cases, although the gradients increase with both *y*H^+^ and ionic strength. There is very little scatter in the data except for those for *y*H^+^ equal to 0.1 in Figure 3a. For this composition some the results for cells 1A and 2A are discordant with other data at 5–15 °C. Also, the results for cells 61 and 62 (solid dots on the plot) were found to disagree with the other data by an almost constant potential at all temperatures, and have been adjusted as noted in Table 5. We show these results, as deviations from an arbitrary fitted line, in Figure 4. The activity coefficients obtained from potentials measured in cells 1, 2, and 7 agree well (as do those from cells 61 and 62 after the adjustment by a fixed Δ*E* noted above). They also have generally lower uncertainties than the other data which are plotted as solid symbols. This illustrates a common feature of the results: differences in the behavior of the pairs of electrodes in the different cells tend to produce changes in potential that vary very little with temperature.

## 5 Conclusions

We have measured cell potentials, and obtained mean activity coefficients of HCl, of aqueous HCl-TrisHCl solutions from 5 to 45 °C and ionic strengths from 1.0 to 5.0 mol kg^–1^. The results agree well with previous studies at 25 °C for ionic strengths 0.1 to 3.0 mol kg^–1^. In combination with other literature data these new measurements should enable a Pitzer ion-interaction model of the solutions to be developed with the particular goal of determining the variation of TrisH^+^-Cl^–^ interaction parameters with temperature. These parameters will be essential components of models of acid–base equilibrium of Tris buffers in NaCl media and in the artificial seawater solutions used to calibrate the seawater total pH scale.

## Supporting Information Available

Description of electrode preparation; determination of standard potentials; densities, water activities, and *p*HCl of the measurement solutions; tabulation of original measured potentials; tabulation of derived γ_HCl_ including uncertainties (PDF)
