# Supplementary material for: Activity Coefficients of HCl in Solutions Related to “Tris” Buffers in Artificial Seawater. I. HCl + TrisHCl + H2O from 1.0 to 5.0 mol kg–1 Ionic Strength, and from 5 to 45 °C
Source: J Chem Eng Data. 2025 Apr 18;70(5):1994–2005. doi: 10.1021/acs.jced.5c00035 (PMC12067379; doi:10.1021/acs.jced.5c00035)
Supplement: Supplementary file 1 — je5c00035_si_001.pdf [file je5c00035_si_001.pdf]

Supporting Information

Activity Coefficients of HCl in Solutions Related to 'Tris' Buffers in Artificial Seawater. I. HCl + TrisHCl + H_2O From 1.0 to 5.0 mol kg^{-1} Ionic Strength, and From 5 ^oC to 45 ^oC

Igor Maksimov,[a*] Toshiaki Asakai, Yuya Hibino, and Simon L. Clegg[b*]

[a] National Metrology Institute of Japan, National Institute of Advanced Industrial Science and Technology (AIST), 1-1-1 Umezono, Tsukuba, Ibaraki 305-8563, Japan
[b] School of Environmental Sciences, University of East Anglia, Norwich NR4 7TJ, United Kingdom

* Corresponding authors. *E-mail*: maksimov.igor@aist.go.jp, and s.clegg@uea.ac.uk

Contents

1. Preparation of the electrodes 2

2. Determination of standard potentials, E^0 4

3. Densities of the solutions 4

4. Estimation of the water activities and *p*HCl of the solutions 5

5. Tabulation of the results 6

6. Tables and charts 7

References 17

This Supporting Information (SI) describes, first of all, the preparation of the electrodes used in the measurements. There are also notes by Andrew Dickson that indicate how practise in his laboratory at Scripps Institution of Oceanography differs from that at NMIJ. Next there is further information, supporting the description in the main text, on the determination of standard potentials of the cells (E^0). We also describe the estimation of the densities of the solutions, their water activities, and equilibrium partial pressures of HCl which are needed in order to adjust cell potentials to a standard 1 atm pressure of H_2.

We tabulate some measured densities of the HCl-TrisHCl solutions from 10 ^oC to 40 ^oC, and these are compared with our model calculations which were used in the adjustment of the cell potentials.

The complete experimental results are tabulated, including the measured potentials, ambient pressures, densities and water activities of the solutions, and the mean activity coefficients of HCl and their estimated uncertainties.

1. Preparation of the electrodes

An NMIJ Harned Cell is shown in Figure S1, below. See the main text of this work for a description of its component parts.

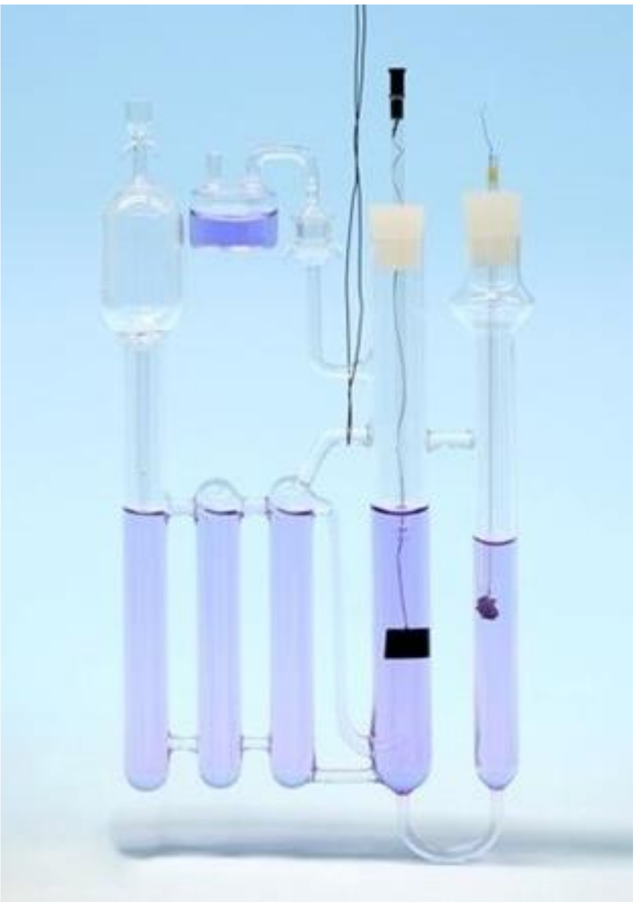

Figure S1. An NMIJ Harned cell. The platinum hydrogen electrode is the dark square in the left half of the U-shaped compartment, and the round shape in the right half is the silver – silver chloride electrode.

The sources and purities of the chemicals used in the preparation of the electrodes are listed in Table S1. The platinized Pt electrodes were made of Pt plates of size 10×10×0.5 mm. Before platinizing, all electrodes are cleaned with hot aqua regia solution and boiling 6 M nitric acid. They were washed after each step with Milli-Q ultrapure water. The platinum black coating is then electrodeposited at a constant current strength of 45 mA, for nine minutes, in 2% chloroplatinic potassium solution with a small addition of lead acetate (50 mg L^{-1}) to improve adhesion. For each measurement run of the test solutions, or 0.01 *m* HCl, freshly prepared sets of platinum hydrogen indicator electrodes are used. The preparation procedure is time-consuming and costly (using 1 gram of Pt reagent for three measurement runs of Harned cells), but in our experience the use of fresh electrodes is necessary to achieve the highest measurement precision.

The silver – silver chloride electrodes of thermal-electrolytic type are prepared as follows: First, pure silver oxide is synthesized by the reaction between silver nitrate of high purity and sodium hydroxide:

$$2NaOH_{(aq)} + 2AgNO_{3(aq)} = 2NaNO_{3(aq)} + Ag_2O_{(s)}\text{ (precipitation)} + H_2O \qquad \text{(S1)}$$

The Ag_2O precipitate is washed thoroughly (at least 40 times) using ultrapure water, and is then stored under humid conditions and in darkness for 6 months before use. This is believed to yield the optimal size of Ag_2O particles.

After such "maturing", a small amount of Ag_2O paste is applied on the spiral Pt coil-wire of the reference electrode being made, and this is then heated at a temperature of 450 ^{o}C for 15 minutes to produce a layer of very pure silver on the platinum support:

$$2\ Ag_2O_{(s)} \rightarrow 4Ag_{(s)} + O_{2(g)} \qquad (S2)$$

This operation of coating and then heating is repeated 4-5 times in order to obtain a silver ball of about 200 mg in weight on the Pt wire.

Finally, ca. 30% of the silver ball is transformed to silver chloride by electrolysis in 1 M ultrapure HCl at a constant current of 10 mA for 40-50 minutes:

$$2Ag_{(s)} + 2HCl_{(aq)} \rightarrow 2AgCl_{(s)} + H_{2(g)} \qquad (S3)$$

The use of ultrapure reagents is essential because any impurity will affect directly the standard potential of the reference electrode. Extreme care is necessary at all steps of silver – silver chloride electrode preparation to avoid unintentional contamination of the silver. Generally, 15-20 electrodes are made in each batch. After the electrolysis the electrodes are stored in a dark place in 0.005 *m* HCl solution for at least one month before use. Electrodes are selected for use in the Harned Cell runs (with 6 individual cells in the water bath) by measuring their potentials in 0.1 *m* NaCl solution after 4 hrs hydrogen bubbling to remove the traces of oxygen. In order to be selected, the six electrodes must have the potentials differing from each other by no more than 10 µV. During the period of use of the electrodes the standard potentials (E^{o}) of the Harned Cells are determined regularly (typically once every two months) to check for consistency and drift. Both the platinum hydrogen and silver – silver chloride electrodes are well rinsed with a portion of the solution to be measured before the electrodes are mounted in the U-shaped compartment shown in Figures 1 and S1 for a measurement run.

When the silver – silver chloride electrodes are stored as noted above (including regularly changing the dilute HCl solution), and are used in measurements of solutions with only a low Cl^- content (and no Br^-), they may last a period of years. Examples of such solutions include pH buffers of up to 0.1 mol kg^{-1} ionic strength. The optimal use of silver – silver chloride electrodes, including determinations of their long-term stability at NMIJ, is described by Maksimov et al.[1]

The procedures for electrode preparation described above are derived from those described by Bates,[2] as are those in other laboratories. Nonetheless there are small differences of detail which may result in differing electrode performance for some solutions, notably those at high ionic strength and Cl^- molality. In Chart 1 we have summarised procedures at the Scripps Institution of Oceanography (Andrew G. Dickson's laboratory) where different from those used in this study.

Maksimov et al.[1] describe the biases in electrode potential that were encountered in the present study (see section 2.2 of the main text), and in related measurements of cell potentials of H^+/$TrisH^+$/Na^+/Cl^- solutions which will be the subject of a future communication. They state that, for the solutions

described in this work, the electrodes behaved normally in the 1.0 mol kg^{-1} ionic strength solutions, showed biases in the 2.0 and 3.0 mol kg^{-1} solutions, and at higher ionic strengths there was significant potential drift and replacement of some electrodes was needed.

2. Determination of standard potentials, E^0

The cells used for measurements of the 0.01 *m* HCl to determine E^0 at each temperature are listed in Table S2, and measured potentials (adjusted to exactly 0.01 m HCl) in Table S3. The results show consistent offsets between individual measurements at the same temperature but on different dates (up to about 0.1 mV at the highest temperatures), reflecting a drift in the potentials and due to use of some electrodes in test solutions containing high molalities of chloride. We have simply averaged the values at each temperature (while omitting a few points that were obviously in error). The uncertainties were calculated, taking account of the drift, as follows.

First, the uncertainties at the temperatures 5, 15, 20 and 25 ^{o}C were calculated using results from cells A-F only. We note that the use of additional results, for cells M-R and S-X would not change noticeably the calculated uncertainties, as the individually determined E^0 agree well. At 10, 30 and 40 ^{o}C the computed uncertainties were based on measurements for cells G-L, and at 35 and 45 ^{o}C on those for cells A1-F1.

In the determinations of the uncertainty of E^0 the standard uncertainties in the temperature, HCl molality and atmospheric pressure were 0.01 K (thermometer certificate), 5×10^{-6} mol kg^{-1} (coulometric titration), and 20 Pa (barometer certificate), respectively. These values were the same for all measurements. However, the standard uncertainties of the measured potentials of the 0.01 *m* HCl solutions were based on the experimental voltage drift during the measurements, and were assigned the following values: 10 μV for the temperatures 5, 10 and 15 ^{o}C, 20 μV for 20, 25 and 30 ^{o}C, 35 μV for 35 ^{o}C, 40 μV for 40 ^{o}C, and 45 μV for 45 ^{o}C.

The uncertainties in E^0 were calculated using standard methods of uncertainty propagation (e.g., JCGM[3]) and are listed in Table S3. As the temperature increases the dominant contribution to the E^0 uncertainty changes from that of the HCl molality (about 77 % at 5 ^{o}C) to that of the cell potential (about 71 % at 45 ^{o}C).

3. Densities of the solutions

The densities of the solutions, at each experimental temperature, are used in the final term in eq 2b for the adjustment of the measured potentials to pH_2 equal to 1 atm. From Table 4 of the main text it can be seen that the effect on the potential of this final term, “JetCorr”, is about 0.02 mV. However, when the HCl mean activity is being calculated the overall influence on γ_{HCl} is that of the difference between the JetCorr terms for the test solution and for the 0.01 *m* HCl used to obtain E^0. This will be a difference of the order of only a few μV.

Densities of the solutions were estimated by assuming additivity of apparent molar volumes of the solutes HCl and TrisHCl in the solution:

$$V_{Tot.} = m\text{TrisHCl}\cdot V^{\Phi}(\text{TrisHCl}) + m\text{HCl}\cdot V^{\Phi}(\text{HCl}) + 1000/\rho(\text{Water}) \quad \text{(S4)}$$

where $V_{Tot.}$ is the estimated total volume of a solution containing 1000 g H_2O, V^{Φ} (cm^3 mol^{-1}) is the apparent molar volume of the indicated solute at its molality (m) in the mixture, and ρ(Water) is the density of pure water in g cm^{-3}. The density of the solution mixture is obtained from the total volume as given above, and the total mass (based upon the same 1000 g of water solvent).

Densities of pure water were obtained from Kell,[4] those of aqueous HCl from Clegg and Wexler,[5] and aqueous TrisHCl solutions from the expression given by Ford et al.[6] and coefficients in their Table 4.

We were also able to measure the densities of the HCl-TrisHCl solutions at 10, 20, 30, and 40 ^{o}C using an Anton Parr digital density meter, model DMA 4100. The calibration certificate of densitometer states that the uncertainties are +/- 0.20 kg m^{-3} at 20 and 25 ^{o}C. It is presumed that they may be greater at other temperatures. The results are given in Table S4.

We compared these measured densities with the calculated ones used for the adjustment of cell potentials, and the differences are summarised in Table S5. They generally increase with total molality, with the exception of the results for $m$$Cl^-$ equal to 3 mol kg^{-1}. For this set of solutions the errors are larger, and the deviations for each value of $y$$H^+$ are grouped together: those for $y$$H^+$ equal to 0.1 all negative, and those for $y$$H^+$ equal to 0.5 all positive. The reason for this is unknown, but is mostly likely experimental error. However, the agreement between measured and calculated values generally is such that errors in our calculated densities are not likely to be significant.

Uncertainties in the densities of the solutions contribute to those of the adjustments of the measured potentials to a pH_2 of 1 atm. We assumed that they are equal, for each total chloride molality, to the mean of the absolute differences between estimated densities, obtained as described above, and those measured in this work (see Table S5). For the case of $m$$Cl^-$ equal to 3 mol kg^{-1}, for which we believe the measurements may be in error, we assumed a value intermediate between those for 2.0 and 4.0 mol kg^{-1} Cl^-. For 1 mol kg^{-1} Cl^-, for which no measurements were made, we assumed the same uncertainty as for $m$$Cl^-$ equal to 2.0 mol kg^{-1}. The values are therefore as follows: ±0.00079 g cm^{-3} ($m$$Cl^-$ = 1.0); ±0.00079 g cm^{-3} ($m$$Cl^-$ = 2.0); ±0.00106 g cm^{-3} ($m$$Cl^-$ = 3.0); ±0.0012 g cm^{-3} ($m$$Cl^-$ = 4.0); and ±0.0019 g cm^{-3} ($m$$Cl^-$ = 5.0).

4. Estimation of the water activities and *p*HCl of the solutions

The water activities of the solutions, at each experimental temperature, are used in the pH_2O term in eq 2b for the adjustment of cell potentials. As noted in the main text, these water activities are calculated using the Pitzer model, with $TrisH^+$-Cl^- parameters from Table 2 of Tishchenko,[7] those for H^+-Cl^- from Holmes et al.,[8] and mixture parameters for H^+-$TrisH^+$ and H^+-$TrisH^+$-Cl^- (known at 25 ^{o}C only) from Bates and Macaskill.[9] Calculated water activities at 5, 25, and 40 ^{o}C for representative solutions are listed in Table S6. The contributions of the pH_2O term to the adjusted values of the cell potentials are largest at the highest measured temperatures. As an illustration, we have also determined equivalent corrections (ΔE) to the cell potentials for cases in which the total pressure P is 1 atm, hence:

$$\Delta E = -RT/(2F) \cdot \ln(1.0 - aH_2O \cdot p^oH_2O) \quad (S5)$$

This simplified case, which neglects the hydrostatic pressure and HCl partial pressure corrections, yields ΔE ranging from 0.087 to 0.100 mV at 5 ^oC, and from 0.85 to 0.99 mV at 40 ^oC for the water activities listed in Table S6. The differences between the corrections at the two temperatures are almost entirely due to the higher water vapour pressure at 40 ^oC. The water activities of the solutions are calculated to vary very little with temperature, and water activities calculated for 25 ^oC (for which Pitzer parameters are generally more accurately known) could have been used at all temperatures without loss of much accuracy. Note that we also calculated water activities at 25 ^oC using $TrisH^+$-Cl^- parameters from Table 2 of Clegg et al.[10] (but with the other parameters the same) and obtained water activities very close to those listed in Table S6 for the same temperature.

Estimates of uncertainties of the water activities were based first of all on the assumption that the modelled water activities at 25 ^oC (for which there is a complete set of Pitzer parameters as noted above) are accurate to an uncertainty equivalent to that of an accurate determination of water activity isopiestically (about 0.0005 in the osmotic coefficient, or ±0.00005 in water activity). Then, for each total chloride molality and temperature, we determined the mean difference between the calculated water activities and the corresponding ones at 25 ^oC. Where the absolute values of these were below 0.00005 we assumed an uncertainty equal to ±0.00005, and where they were greater we assumed values equal to 0.00005 plus 15% of the difference between the two. While somewhat arbitrary, this procedure accounts for the fact that calculated water activities at temperatures other than 25 ^oC will have greater uncertainties, related to the availability and confidence in the available Pitzer model parameters. Our estimated uncertainties in the water activities are listed in Table S7.

Equilibrium partial pressures of HCl above the measurement solutions were calculated using this expression:

$$pHCl = aH^+ \cdot aCl^-/K_H \quad (S6)$$

where K_H ($mol^2\ kg^{-2}\ atm^{-1}$) is the Henry's law constant of HCl at the temperature of interest, given by:

$$\ln(K_H) = 4.6187 + 5977.5014/T - 0.03401 \cdot T \quad (S7)$$

This expression approximates very closely the values of the Henry's law constant of HCl given in Table II of Brimblecombe and Clegg.[11] The highest pHCl occurs for the 5 mol kg^{-1} chloride solution for which $yH^+ = 0.5$, and is about 0.45×10^{-4} atm at 40 ^oC, which corresponds to a change of only about 0.7×10^{-3} mV in the adjustment of a measured potential. Because of this very small magnitude we did not estimate uncertainties of the partial pressures.

5. Tabulation of the results

Complete experimental results are listed in Table S8. This contains the pressure, P, for each measurement; cell potentials including the original measured values (before the correction to pH_2 equal to 1 atm); the estimated uncertainties of the HCl mean activity coefficient obtained from the measurements; and the water activity and density of each solution (used in the pressure correction).

6. Tables and Charts

Chart S1. Procedures for electrode preparation and use at Scripps Institution of Oceanography,[a] where different from those at NMIJ.

**Sequential steps in electrode preparation and use**	**Notes regarding the procedures used at Scripps Institution of Oceanography , originating from methods described by Bates[2]**
Cleaning of the Pt hydrogen electrodes	We clean with *aqua regia* alone (and then rinse with water).
Electro-deposition of Pt black coating	We use chloroplatinic acid (that we prepare ourselves), not a potassium salt. The electrolysis time / current density is similar.
Storage of Ag_2O	We store the Ag_2O under water, though we do first use it sooner than 6 months later. We partially dry a small amount to a consistency similar to that of toothpaste, before thermally decomposing it to Ag as part of the electrode.
Application of Ag_2O paste to spiral Pt wire	We first heat the Pt wire in an alcohol flame to clean it before use. It is heated to a red glow.<br><br>After application of the paste we heat initially for 15 min at 100 °C to dry the Ag_2O , then raise the temperature to 495 °C for a further 15 min; the oven is then turned off and allowed to cool for 45 min with the electrodes still in it. (Note: the glass we use for the electrodes is soda-glass to ensure a good seal around the Pt wire, so this care is necessary.)<br><br>These (partially prepared) electrodes are stored overnight in water before being chloridized.
Chloridisation of the electrodes (equation S3)	We chloridize 7 electrodes at once (thus 70 mA in all, for 45 min). Our set of 7 electrodes is then stored in 0.01 *m* HCl for at least 48 h, while they are all electrically connected to each other.
Selection of the chloride electrodes	We measure the *bias* potentials between our set of 7 electrodes, and select those 6 that agree most closely. We choose 6, because (in the same way as NMIJ) we can run a maximum of 6 Harned cells at once in our bath.
Use of the chloride electrodes	We will usually only use such electrodes once, they are then cleaned (in hot concentrated HNO_3) to remove the Ag, prior to reusing the electrode bases to make fresh electrodes.

[a] This information was provided by Andrew G. Dickson (adickson@ucsd.edu).

Table S1. Reagents used to prepare platinum hydrogen electrodes, and silver – silver chloride reference electrodes

Chemical	CAS Registry #	Molar mass	Supplier or source	Notes
HNO_3 (used for aqua regia)	7697-37-2	63.0129 g	FUJIFILM WAKO Pure Chemical Corp.	The manufacturer's certificate stated that the concentration was 60.0 mass %, and this value was assumed.
HCl (used for aqua regia)	7647-01-0	36.4609 g	FUJIFILM WAKO Pure Chemical Corp.	The manufacturer's certificate statement of concentration 35.0 mass %
HCl (used for electrolysis)	7647-01-0	36.4609 g	Kanto Chemical Co.	Ultrapure grade aqueous HCl of 31.4 mass % (diluted with water and then molality determined before use).
NaOH	1310-73-2	39.9971 g	FUJIFILM WAKO Pure Chemical Corp.	The manufacturer's certificate statement of purity 99.7 mass %
$AgNO_3$	7761-88-8	169.873 g	Thermo Fisher Scientific, USA	The manufacturer's certificate statement of purity 99.995 mass %
$K_2(PtCl_4)$	10025-99-7	415.088 g	FUJIFILM WAKO Pure Chemical Corp.	The manufacturer's certificate statement of purity 44 mass % (as Pt)
$(CH_3COO)_2Pb·3H_2O$	6080-56-4	379.344 g	FUJIFILM WAKO Pure Chemical Corp.	The manufacturer's certificate statement of purity 99.7 mass %
H_2O	7732-18-5	18.0153 g	Milli-Q Ultra Pure Water System (Merck)	Resistivity 18.2 MΩ cm at 25 °C

Table S2. Cells Used to Measure 0.01 *m* HCl at Each Temperature [a]

t (°C)	Cells		*t* (°C)	Cells
5	A–F, M–R, S–X		30	G–L, M–R, S–X
10	G–L, M–R, S–X		35	A1–F1
15	A–F, M–R, S–X, A1–F1		40	G–L, M–R, S–X
20	A–F, M–R, S–X		45	A1–F1
25	A–F, M–R, S–X			

[a] See Table 3 in the main text for the dates of the measurements.

Table S3. Standard potentials (E^0), and their uncertainties (u) determined in this work

t (°C)	E^0 (V)	$u(E^0)$ (V)	t (°C)	E^0 (V)	$u(E^0)$ (V)
5	0.234076	0.000027	30	0.219192	0.000034
10	0.231422	0.000028	35	0.215696	0.000045
15	0.228573	0.000028	40	0.212104	0.000057
20	0.225610	0.000033	45	0.208306	0.000060
25	0.222463	0.000034			

Table S4. Measured densities (kg m^{-3}) of studied solutions at from 10 to 40 °C

t (°C)	yH$^+$ = 0.1	yH$^+$ = 0.3	yH$^+$ = 0.5
	mCl$^-$ = 2 mol kg^{-1}		
10	1079.58	1070.65	1061.03
20	1076.92	1067.95	1058.44
30	1073.53	1064.58	1055.13
40	1069.54	1060.72	1051.29
	mCl$^-$ = 3 mol kg^{-1}		
10	1102.09	1096.32	1089.79
20	1098.91	1093.23	1087.05
30	1095.28	1089.63	1083.46
40	1091.24	1085.41	1079.30
	mCl$^-$ = 4 mol kg^{-1}		
10	1132.68	1121.31	1107.41
20	1129.26	1117.86	1103.95
30	1125.41	1114.02	1100.10
40	1121.19	1109.81	1095.91
	mCl$^-$ = 5 mol kg^{-1}		
10	1154.64	1141.44	1126.58
20	1150.89	1137.78	1122.90
30	1146.88	1133.73	1118.83
40	1142.57	1129.43	1114.57

Table S5. Differences between measured and estimated densities of the solution mixtures at each chloride molality

mCl$^-$ (mol kg^{-1})	Mean absolute deviation^a (g cm^{-3})	Max. deviation (g cm^{-3})	Min. deviation (g cm^{-3})
2.0	0.00079	0.0021	-0.00036
3.0	0.0033 (see note in text)	0.0048	-0.0063
4.0	0.0012	0.0035	-0.0015
5.0	0.0019	0.0057	-0.0022

^{a} This is the absolute value of measured minus the calculated density, for all temperatures, and for all H$^+$ cation fractions.

Table S6. Calculated water activities of some of the solutions[a]

mCl$^-$ (mol kg^{-1})	aH$_2$O (5 °C)	aH$_2$O (25 °C)	aH$_2$O (40 °C)
1.0	0.9681	0.9681	0.9681
2.0	0.9363	0.9361	0.9362
3.0	0.9044	0.9039	0.9040
4.0	0.8730	0.8720	0.8722
5.0	0.8425	0.8409	0.8413

[a] The value of yH$^+$ is equal to 0.1 in all cases.

Table S7. Estimated uncertainties in the water activities at each total Cl$^-$ molality and temperature [a]

t (°C)	Estimated uncertainty in water activity at the indicated Cl$^-$ molality				
	1.0 mol kg^{-1}	2.0 mol kg^{-1}	3.0 mol kg^{-1}	4.0 mol kg^{-1}	5.0 mol kg^{-1}
5	0.000054	0.000148	0.000253	0.000384	0.000677
10	0.000073	0.000122	0.000208	0.000325	0.000522
15	0.000062	0.000092	0.000162	0.000245	0.000432
20	0.000058	0.00008	0.000118	0.000166	0.000296
25	0.00005	0.00005	0.00005	0.00005	0.00005
30	0.000054	0.00005	0.00005	0.000068	0.000123
35	0.00005				
40	0.00005	0.000073	0.000092	0.000083	0.00005
45	0.00005				

[a] There are no measurements of potential for the temperatures and chloride molalities for which the entries are blank.

Table S8. Harned Cell Results [a]

Cell	t (°C)[b]	P (atm)	mCl$^-$ (mol kg^{-1})	yH$^+$	mHCl (mol kg^{-1})	mTrisHCl (mol kg^{-1})	E(meas.) (V)[c]	E (V)[c]	E(adj.) (V)[c]	u(E) (mV)	γ_{HCl}	u(γ_{HCl})	aH$_2$O	ρ (g cm^{-3})
1A	5	0.998204	1.0	0.10	0.10001	0.90027	0.304921	0.305028	0.305032	0.144	0.71966	0.00217	0.9681	1.0442
2A	5	0.998204	1.0	0.10	0.10001	0.90025	0.304569	0.304676	0.304680	0.144	0.72497	0.00219	0.9681	1.0442
3A	5	0.998204	1.0	0.30	0.30010	0.70020	0.276694	0.276801	0.276805	0.010	0.74859	0.00022	0.9669	1.0390
4A	5	0.998204	1.0	0.30	0.30011	0.70018	0.276693	0.276800	0.276804	0.010	0.74859	0.00022	0.9669	1.0390
5A	5	0.998204	1.0	0.50	0.50016	0.50011	0.262967	0.263074	0.263078	0.010	0.77211	0.00022	0.9658	1.0334
6A	5	0.998204	1.0	0.50	0.50013	0.50013	0.262965	0.263072	0.263076	0.010	0.77217	0.00022	0.9658	1.0334
1A	10	0.995884	1.0	0.10	0.10001	0.90027	0.303756	0.303936	0.303914	0.141	0.71544	0.00208	0.9681	1.0434
2A	10	0.995884	1.0	0.10	0.10001	0.90025	0.303412	0.303592	0.303570	0.141	0.72051	0.00209	0.9681	1.0434
3A	10	0.995884	1.0	0.30	0.30010	0.70020	0.275051	0.275231	0.275209	0.010	0.74376	0.00023	0.9669	1.0382
4A	10	0.995884	1.0	0.30	0.30011	0.70018	0.275051	0.275231	0.275209	0.010	0.74374	0.00023	0.9669	1.0382
5A	10	0.995884	1.0	0.50	0.50016	0.50011	0.261099	0.261279	0.261257	0.010	0.76679	0.00024	0.9658	1.0327
6A	10	0.995884	1.0	0.50	0.50013	0.50013	0.261097	0.261277	0.261255	0.010	0.76684	0.00024	0.9658	1.0327
1A	15	0.995934	1.0	0.10	0.10001	0.90027	0.302464	0.302704	0.302691	0.118	0.71061	0.00169	0.9680	1.0423
2A	15	0.995934	1.0	0.10	0.10001	0.90025	0.302125	0.302365	0.302352	0.118	0.71548	0.00170	0.9680	1.0423
3A	15	0.995934	1.0	0.30	0.30010	0.70020	0.273264	0.273504	0.273491	0.061	0.73856	0.00091	0.9670	1.0372
4A	15	0.995934	1.0	0.30	0.30011	0.70018	0.273262	0.273502	0.273489	0.061	0.73858	0.00091	0.9670	1.0372
5A	15	0.995934	1.0	0.50	0.50016	0.50011	0.259098	0.259338	0.259325	0.060	0.76093	0.00092	0.9659	1.0317
6A	15	0.995934	1.0	0.50	0.50013	0.50013	0.259096	0.259336	0.259323	0.060	0.76099	0.00092	0.9659	1.0317
1	15	0.994355	1.0	0.10	0.10004	0.90020	0.302201	0.302461	0.302448	0.118	0.71403	0.00170	0.9680	1.0423
2	15	0.994355	1.0	0.10	0.10000	0.90026	0.302202	0.302462	0.302449	0.118	0.71412	0.00170	0.9680	1.0423
3	15	0.994355	1.0	0.30	0.30006	0.70020	0.273343	0.273603	0.273590	0.061	0.73715	0.00091	0.9670	1.0372
4	15	0.994355	1.0	0.30	0.30007	0.70018	0.273351	0.273611	0.273598	0.061	0.73702	0.00091	0.9670	1.0372
5	15	0.994355	1.0	0.50	0.50007	0.50012	0.259159	0.259419	0.259406	0.060	0.75980	0.00092	0.9659	1.0317
6	15	0.994355	1.0	0.50	0.50015	0.50014	0.259016	0.259276	0.259263	0.060	0.76188	0.00092	0.9659	1.0317
7	20	0.991009	1.0	0.10	0.10004	0.90021	0.300698	0.301084	0.301045	0.137	0.70971	0.00194	0.9680	1.0410
8	20	0.991009	1.0	0.10	0.10004	0.90025	0.300576	0.300962	0.300923	0.137	0.71140	0.00194	0.9680	1.0410
9	20	0.991009	1.0	0.30	0.30009	0.70021	0.271312	0.271698	0.271658	0.010	0.73306	0.00026	0.9670	1.0359
10	20	0.991009	1.0	0.30	0.30008	0.70017	0.271314	0.271700	0.271660	0.010	0.73306	0.00026	0.9670	1.0359
11	20	0.991009	1.0	0.50	0.50016	0.50011	0.256943	0.257329	0.257289	0.011	0.75463	0.00027	0.9659	1.0305
12	20	0.991009	1.0	0.50	0.50017	0.50012	0.256936	0.257322	0.257282	0.011	0.75472	0.00027	0.9659	1.0305
1	25	0.993654	1.0	0.10	0.10004	0.90020	0.299179	0.299642	0.299579	0.077	0.70400	0.00106	0.9681	1.0395
2	25	0.993654	1.0	0.10	0.10000	0.90026	0.299178	0.299641	0.299578	0.077	0.70412	0.00106	0.9681	1.0395
3	25	0.993654	1.0	0.30	0.30006	0.70020	0.269359	0.269822	0.269758	0.020	0.72624	0.00029	0.9670	1.0344
4	25	0.993654	1.0	0.30	0.30007	0.70018	0.269360	0.269823	0.269759	0.020	0.72621	0.00029	0.9670	1.0344
5	25	0.993654	1.0	0.50	0.50007	0.50012	0.254699	0.255162	0.255098	0.099	0.74832	0.00144	0.9660	1.0290
6	25	0.993654	1.0	0.50	0.50015	0.50014	0.254562	0.255025	0.254961	0.099	0.75021	0.00145	0.9660	1.0291
7	30	0.990654	1.0	0.10	0.10004	0.90021	0.297374	0.298026	0.297934	0.101	0.69913	0.00136	0.9681	1.0378
8	30	0.990654	1.0	0.10	0.10004	0.90025	0.297241	0.297893	0.297801	0.101	0.70088	0.00137	0.9681	1.0378
9	30	0.990654	1.0	0.30	0.30009	0.70021	0.267000	0.267652	0.267560	0.020	0.72193	0.00032	0.9671	1.0328
10	30	0.990654	1.0	0.30	0.30008	0.70017	0.267005	0.267657	0.267565	0.020	0.72189	0.00032	0.9671	1.0328
11	30	0.990654	1.0	0.50	0.50016	0.50011	0.252129	0.252780	0.252688	0.020	0.74335	0.00033	0.9660	1.0274
12	30	0.990654	1.0	0.50	0.50017	0.50012	0.252129	0.252780	0.252688	0.020	0.74333	0.00033	0.9660	1.0274
1	35	0.993121	1.0	0.10	0.10004	0.90020	0.295474	0.296288	0.296252	0.020	0.69316	0.00026	0.9681	1.0360
2	35	0.993121	1.0	0.10	0.10000	0.90026	0.295475	0.296289	0.296253	0.020	0.69325	0.00026	0.9681	1.0360
3	35	0.993121	1.0	0.30	0.30006	0.70020	0.264700	0.265513	0.265477	0.020	0.71444	0.00027	0.9671	1.0310
4	35	0.993121	1.0	0.30	0.30007	0.70018	0.264695	0.265508	0.265472	0.020	0.71449	0.00027	0.9671	1.0310
5	35	0.993121	1.0	0.50	0.50007	0.50012	0.249587	0.250399	0.250363	0.022	0.73563	0.00031	0.9661	1.0256
6	35	0.993121	1.0	0.50	0.50015	0.50014	0.249440	0.250252	0.250216	0.022	0.73757	0.00031	0.9661	1.0257
7	40	0.990368	1.0	0.10	0.10004	0.90021	0.293328	0.294438	0.294404	0.150	0.68757	0.00197	0.9681	1.0340
8	40	0.990368	1.0	0.10	0.10004	0.90025	0.293178	0.294288	0.294254	0.150	0.68946	0.00197	0.9681	1.0340

9	40	0.990368	1.0	0.30	0.30009	0.70021	0.262008	0.263117	0.263083	0.040	0.70927	0.00071	0.9672	1.0290
10	40	0.990368	1.0	0.30	0.30008	0.70017	0.262012	0.263121	0.263087	0.040	0.70924	0.00071	0.9672	1.0290
11	40	0.990368	1.0	0.50	0.50016	0.50011	0.246612	0.247720	0.247686	0.040	0.73078	0.00073	0.9662	1.0237
12	40	0.990368	1.0	0.50	0.50017	0.50012	0.246618	0.247726	0.247692	0.040	0.73069	0.00073	0.9662	1.0237
1	45	0.993753	1.0	0.10	0.10004	0.90020	0.291048	0.292443	0.292476	0.040	0.68151	0.00051	0.9682	1.0318
2	45	0.993753	1.0	0.10	0.10000	0.90026	0.291048	0.292443	0.292476	0.040	0.68161	0.00051	0.9682	1.0318
3	45	0.993753	1.0	0.30	0.30006	0.70020	0.259313	0.260706	0.260740	0.083	0.70196	0.00107	0.9672	1.0269
4	45	0.993753	1.0	0.30	0.30007	0.70018	0.259210	0.260603	0.260637	0.083	0.70327	0.00107	0.9672	1.0269
5	45	0.993753	1.0	0.50	0.50007	0.50012	0.243771	0.245163	0.245197	0.123	0.72198	0.00163	0.9663	1.0216
6	45	0.993753	1.0	0.50	0.50015	0.50014	0.243605	0.244997	0.245031	0.123	0.72406	0.00163	0.9663	1.0216
61	5	0.991443	1.0	0.10	0.10003	0.90026	0.304683	0.304744	0.304747	0.144	0.72389	0.00218	0.9681	1.0442
62	5	0.991443	1.0	0.10	0.10005	0.90022	0.304677	0.304737	0.304741	0.144	0.72389	0.00218	0.9681	1.0442
61	10	0.989035	1.0	0.10	0.10003	0.90026	0.303519	0.303655	0.303633	0.141	0.71952	0.00209	0.9681	1.0434
62	10	0.989035	1.0	0.10	0.10005	0.90022	0.303512	0.303648	0.303626	0.141	0.71953	0.00209	0.9681	1.0434
61	15	0.990476	1.0	0.10	0.10003	0.90026	0.302264	0.302444	0.302431	0.118	0.71429	0.00170	0.9680	1.0423
62	15	0.990476	1.0	0.10	0.10005	0.90022	0.302256	0.302436	0.302423	0.118	0.71431	0.00170	0.9680	1.0423
61	20	1.001550	1.0	0.10	0.10003	0.90026	0.300998	0.301119	0.301079	0.137	0.70927	0.00194	0.9680	1.0410
62	20	1.001550	1.0	0.10	0.10005	0.90022	0.300989	0.301110	0.301070	0.137	0.70930	0.00194	0.9680	1.0410
61	25	1.001352	1.0	0.10	0.10003	0.90026	0.299414	0.299646	0.299583	0.077	0.70396	0.00106	0.9681	1.0395
62	25	1.001352	1.0	0.10	0.10005	0.90022	0.299405	0.299637	0.299574	0.077	0.70399	0.00106	0.9681	1.0395
61	30	1.000464	1.0	0.10	0.10003	0.90026	0.297652	0.298041	0.297949	0.101	0.69897	0.00136	0.9681	1.0378
62	30	1.000464	1.0	0.10	0.10005	0.90022	0.297642	0.298031	0.297939	0.101	0.69901	0.00136	0.9681	1.0378
61	40	1.001372	1.0	0.10	0.10003	0.90026	0.293646	0.294467	0.294433	0.150	0.68725	0.00197	0.9681	1.0340
62	40	1.001372	1.0	0.10	0.10005	0.90022	0.293636	0.294457	0.294423	0.150	0.68729	0.00197	0.9681	1.0340
13	5	0.994957	2.0	0.10	0.20008	1.80102	0.267233	0.267376	0.267379	0.010	0.78902	0.00023	0.9363	1.0802
14	5	0.994957	2.0	0.10	0.20009	1.80101	0.267232	0.267375	0.267378	0.010	0.78901	0.00023	0.9363	1.0802
15	5	0.994957	2.0	0.30	0.60024	1.40047	0.237892	0.238035	0.238038	0.010	0.84021	0.00024	0.9323	1.0718
16	5	0.994957	2.0	0.30	0.60033	1.40081	0.237894	0.238037	0.238040	0.010	0.84002	0.00024	0.9323	1.0718
17	5	0.994957	2.0	0.50	1.00058	1.00060	0.222612	0.222754	0.222758	0.010	0.89496	0.00026	0.9280	1.0626
18	5	0.994957	2.0	0.50	1.00048	1.00052	0.222611	0.222753	0.222757	0.010	0.89506	0.00026	0.9280	1.0626
19	10	0.973718	2.0	0.10	0.20011	1.80096	0.265517	0.265969	0.265947	0.010	0.77854	0.00024	0.9362	1.0789
20	10	0.973718	2.0	0.10	0.20011	1.80100	0.265515	0.265967	0.265945	0.010	0.77856	0.00024	0.9362	1.0789
21	10	0.973718	2.0	0.30	0.60037	1.40075	0.235463	0.235915	0.235893	0.011	0.83209	0.00027	0.9323	1.0706
22	10	0.973718	2.0	0.30	0.60034	1.40073	0.235467	0.235919	0.235897	0.011	0.83206	0.00027	0.9323	1.0706
23	10	0.973718	2.0	0.50	1.00056	1.00057	0.219995	0.220446	0.220424	0.011	0.88495	0.00029	0.9282	1.0614
24	10	0.973718	2.0	0.50	1.00057	1.00052	0.219991	0.220442	0.220420	0.011	0.88503	0.00029	0.9282	1.0614
13	15	0.993585	2.0	0.10	0.20008	1.80102	0.263798	0.264060	0.264047	0.010	0.77343	0.00017	0.9361	1.0774
14	15	0.993585	2.0	0.10	0.20009	1.80101	0.263796	0.264058	0.264045	0.010	0.77344	0.00017	0.9361	1.0774
15	15	0.993585	2.0	0.30	0.60024	1.40047	0.233454	0.233716	0.233703	0.010	0.82276	0.00018	0.9324	1.0691
16	15	0.993585	2.0	0.30	0.60033	1.40081	0.233456	0.233718	0.233705	0.010	0.82258	0.00018	0.9324	1.0691
17	15	0.993585	2.0	0.50	1.00058	1.00060	0.217722	0.217983	0.217970	0.010	0.87467	0.00019	0.9283	1.0600
18	15	0.993585	2.0	0.50	1.00048	1.00052	0.217720	0.217981	0.217968	0.010	0.87478	0.00019	0.9283	1.0600
13	20	0.993496	2.0	0.10	0.20008	1.80102	0.261886	0.262230	0.262190	0.011	0.76556	0.00028	0.9361	1.0757
14	20	0.993496	2.0	0.10	0.20009	1.80101	0.261883	0.262227	0.262187	0.011	0.76558	0.00028	0.9361	1.0757
15	20	0.993496	2.0	0.30	0.60024	1.40047	0.231047	0.231390	0.231350	0.011	0.81388	0.00029	0.9325	1.0675
16	20	0.993496	2.0	0.30	0.60033	1.40081	0.231051	0.231394	0.231354	0.011	0.81367	0.00029	0.9324	1.0675
17	20	0.993496	2.0	0.50	1.00058	1.00060	0.215078	0.215420	0.215380	0.011	0.86461	0.00031	0.9285	1.0585
18	20	0.993496	2.0	0.50	1.00048	1.00052	0.215075	0.215417	0.215377	0.011	0.86475	0.00031	0.9285	1.0585
13	25	0.995115	2.0	0.10	0.20008	1.80102	0.259843	0.260273	0.260210	0.020	0.75719	0.00030	0.9361	1.0738
14	25	0.995115	2.0	0.10	0.20009	1.80101	0.259840	0.260270	0.260207	0.020	0.75721	0.00030	0.9361	1.0738
15	25	0.995115	2.0	0.30	0.60024	1.40047	0.228511	0.228940	0.228876	0.020	0.80447	0.00032	0.9325	1.0657
16	25	0.995115	2.0	0.30	0.60033	1.40081	0.228515	0.228944	0.228880	0.020	0.80426	0.00032	0.9325	1.0657
17	25	0.995115	2.0	0.50	1.00058	1.00060	0.212350	0.212777	0.212714	0.021	0.85329	0.00035	0.9287	1.0567
18	25	0.995115	2.0	0.50	1.00048	1.00052	0.212344	0.212771	0.212708	0.021	0.85347	0.00035	0.9287	1.0567

19	30	0.971675	2.0	0.10	0.20011	1.80096	0.257520	0.258416	0.258324	0.021	0.74592	0.00035	0.9361	1.0718
20	30	0.971675	2.0	0.10	0.20011	1.80100	0.257512	0.258408	0.258316	0.021	0.74602	0.00035	0.9361	1.0718
21	30	0.971675	2.0	0.30	0.60037	1.40075	0.225502	0.226396	0.226304	0.020	0.79483	0.00036	0.9326	1.0638
22	30	0.971675	2.0	0.30	0.60034	1.40073	0.225505	0.226399	0.226307	0.020	0.79481	0.00036	0.9326	1.0638
23	30	0.971675	2.0	0.50	1.00056	1.00057	0.209073	0.209965	0.209873	0.020	0.84322	0.00038	0.9289	1.0549
24	30	0.971675	2.0	0.50	1.00057	1.00052	0.209069	0.209961	0.209869	0.020	0.84328	0.00038	0.9289	1.0549
19	40	0.971981	2.0	0.10	0.20011	1.80096	0.252563	0.253910	0.253876	0.081	0.72833	0.00120	0.9362	1.0674
20	40	0.971981	2.0	0.10	0.20011	1.80100	0.252462	0.253809	0.253775	0.081	0.72969	0.00120	0.9362	1.0674
21	40	0.971981	2.0	0.30	0.60037	1.40075	0.219547	0.220891	0.220856	0.041	0.77527	0.00078	0.9329	1.0596
22	40	0.971981	2.0	0.30	0.60034	1.40073	0.219555	0.220899	0.220864	0.041	0.77518	0.00078	0.9329	1.0596
23	40	0.971981	2.0	0.50	1.00056	1.00057	0.202669	0.204009	0.203975	0.040	0.82108	0.00082	0.9293	1.0508
24	40	0.971981	2.0	0.50	1.00057	1.00052	0.202663	0.204003	0.203969	0.040	0.82117	0.00082	0.9293	1.0508
31	5	0.990703	3.0	0.10	0.30024	2.70224	0.242602	0.242793	0.242796	0.010	0.87813	0.00025	0.9044	1.1102
32	5	0.990703	3.0	0.10	0.30023	2.70228	0.242603	0.242794	0.242797	0.010	0.87812	0.00025	0.9044	1.1102
33	5	0.990703	3.0	0.30	0.90080	2.10180	0.211743	0.211933	0.211937	0.010	0.96504	0.00028	0.8962	1.1000
34	5	0.990703	3.0	0.30	0.90078	2.10176	0.211745	0.211935	0.211939	0.010	0.96502	0.00028	0.8962	1.1000
35	5	0.990703	3.0	0.50	1.50131	1.50127	0.194662	0.194851	0.194855	0.010	1.06752	0.00031	0.8871	1.0883
36	5	0.990703	3.0	0.50	1.50121	1.50126	0.194663	0.194852	0.194856	0.010	1.06756	0.00031	0.8871	1.0883
25	10	0.983518	3.0	0.10	0.30016	2.70218	0.240520	0.240843	0.240821	0.010	0.86846	0.00027	0.9043	1.1084
26	10	0.983518	3.0	0.10	0.30032	2.70213	0.240520	0.240843	0.240821	0.010	0.86820	0.00027	0.9043	1.1084
27	10	0.983518	3.0	0.30	0.90074	2.10170	0.209182	0.209504	0.209482	0.087	0.95284	0.00172	0.8963	1.0983
28	10	0.983518	3.0	0.30	0.90078	2.10181	0.209304	0.209626	0.209604	0.087	0.95041	0.00171	0.8963	1.0983
29	10	0.983518	3.0	0.50	1.50117	1.50114	0.191877	0.192198	0.192176	0.090	1.05227	0.00196	0.8875	1.0867
30	10	0.983518	3.0	0.50	1.50114	1.50117	0.191750	0.192071	0.192049	0.090	1.05503	0.00196	0.8875	1.0867
31	15	0.990180	3.0	0.10	0.30024	2.70224	0.238573	0.238871	0.238858	0.010	0.85599	0.00019	0.9041	1.1065
32	15	0.990180	3.0	0.10	0.30023	2.70228	0.238572	0.238871	0.238857	0.010	0.85601	0.00019	0.9041	1.1065
33	15	0.990180	3.0	0.30	0.90080	2.10180	0.206695	0.206992	0.206979	0.010	0.93900	0.00021	0.8964	1.0964
34	15	0.990180	3.0	0.30	0.90078	2.10176	0.206696	0.206993	0.206980	0.010	0.93900	0.00021	0.8964	1.0964
35	15	0.990180	3.0	0.50	1.50131	1.50127	0.189200	0.189495	0.189482	0.011	1.03456	0.00025	0.8878	1.0849
36	15	0.990180	3.0	0.50	1.50121	1.50126	0.189196	0.189491	0.189478	0.011	1.03470	0.00025	0.8878	1.0849
31	20	0.989983	3.0	0.10	0.30024	2.70224	0.236374	0.236754	0.236714	0.011	0.84477	0.00031	0.9039	1.1044
32	20	0.989983	3.0	0.10	0.30023	2.70228	0.236371	0.236751	0.236711	0.011	0.84482	0.00031	0.9039	1.1044
33	20	0.989983	3.0	0.30	0.90080	2.10180	0.203992	0.204370	0.204330	0.010	0.92580	0.00032	0.8965	1.0944
34	20	0.989983	3.0	0.30	0.90078	2.10176	0.203994	0.204372	0.204332	0.010	0.92578	0.00032	0.8965	1.0944
35	20	0.989983	3.0	0.50	1.50131	1.50127	0.186302	0.186677	0.186637	0.012	1.01784	0.00038	0.8882	1.0830
36	20	0.989983	3.0	0.50	1.50121	1.50126	0.186294	0.186669	0.186629	0.012	1.01805	0.00038	0.8882	1.0830
31	25	0.991404	3.0	0.10	0.30024	2.70224	0.234047	0.234513	0.234449	0.020	0.83309	0.00033	0.9039	1.1021
32	25	0.991404	3.0	0.10	0.30023	2.70228	0.234045	0.234511	0.234447	0.020	0.83312	0.00033	0.9039	1.1022
33	25	0.991404	3.0	0.30	0.90080	2.10180	0.201179	0.201642	0.201578	0.020	0.91184	0.00036	0.8967	1.0923
34	25	0.991404	3.0	0.30	0.90078	2.10176	0.201180	0.201643	0.201579	0.020	0.91184	0.00036	0.8967	1.0923
35	25	0.991404	3.0	0.50	1.50131	1.50127	0.183278	0.183738	0.183674	0.080	1.00073	0.00156	0.8886	1.0810
36	25	0.991404	3.0	0.50	1.50121	1.50126	0.183169	0.183629	0.183565	0.080	1.00291	0.00156	0.8886	1.0810
25	30	0.983212	3.0	0.10	0.30016	2.70218	0.231426	0.232143	0.232051	0.020	0.82214	0.00037	0.9039	1.0998
26	30	0.983212	3.0	0.10	0.30032	2.70213	0.231421	0.232138	0.232046	0.020	0.82197	0.00037	0.9039	1.0998
27	30	0.983212	3.0	0.30	0.90074	2.10170	0.198106	0.198819	0.198727	0.020	0.89808	0.00040	0.8969	1.0901
28	30	0.983212	3.0	0.30	0.90078	2.10181	0.198106	0.198819	0.198727	0.020	0.89804	0.00040	0.8969	1.0901
29	30	0.983212	3.0	0.50	1.50117	1.50114	0.179855	0.180563	0.180471	0.085	0.98662	0.00162	0.8890	1.0789
30	30	0.983212	3.0	0.50	1.50114	1.50117	0.179737	0.180446	0.180353	0.085	0.98887	0.00163	0.8890	1.0789
25	40	0.983647	3.0	0.10	0.30016	2.70218	0.225964	0.227103	0.227069	0.040	0.79781	0.00079	0.9040	1.0949
26	40	0.983647	3.0	0.10	0.30032	2.70213	0.225961	0.227100	0.227066	0.040	0.79762	0.00079	0.9040	1.0949
27	40	0.983647	3.0	0.30	0.90074	2.10170	0.191686	0.192818	0.192784	0.040	0.86927	0.00086	0.8974	1.0855
28	40	0.983647	3.0	0.30	0.90078	2.10181	0.191683	0.192815	0.192781	0.040	0.86928	0.00086	0.8974	1.0855
29	40	0.983647	3.0	0.50	1.50117	1.50114	0.172899	0.174024	0.173989	0.041	0.95386	0.00096	0.8899	1.0746
30	40	0.983647	3.0	0.50	1.50114	1.50117	0.172889	0.174014	0.173979	0.041	0.95406	0.00096	0.8899	1.0746

37	5	0.992884	4.0	0.10	0.40025	3.60202	0.223713	0.223874	0.223877	0.012	0.97749	0.00031	0.8730	1.1354
38	5	0.992884	4.0	0.10	0.40021	3.60218	0.223704	0.223865	0.223868	0.012	0.97770	0.00031	0.8730	1.1354
39	5	0.992884	4.0	0.30	1.20070	2.80167	0.191206	0.191365	0.191369	0.010	1.11189	0.00032	0.8595	1.1242
40	5	0.992884	4.0	0.30	1.20067	2.80165	0.191205	0.191364	0.191368	0.010	1.11194	0.00032	0.8595	1.1242
41	5	0.992884	4.0	0.50	2.00127	2.00128	0.172285	0.172443	0.172447	0.014	1.27804	0.00045	0.8441	1.1110
42	5	0.992884	4.0	0.50	2.00122	2.00118	0.172294	0.172452	0.172456	0.014	1.27784	0.00045	0.8441	1.1110
43	10	0.990822	4.0	0.10	0.40022	3.60222	0.221454	0.221681	0.221659	0.011	0.96466	0.00032	0.8727	1.1332
44	10	0.990822	4.0	0.10	0.40017	3.60199	0.221449	0.221676	0.221654	0.011	0.96486	0.00032	0.8727	1.1332
45	10	0.990822	4.0	0.30	1.20075	2.80174	0.188403	0.188628	0.188606	0.070	1.09635	0.00160	0.8597	1.1221
46	10	0.990822	4.0	0.30	1.20071	2.80166	0.188557	0.188782	0.188760	0.070	1.09293	0.00159	0.8597	1.1221
47	10	0.990822	4.0	0.50	2.00123	2.00116	0.169339	0.169562	0.169540	0.017	1.25519	0.00053	0.8447	1.1090
48	10	0.990822	4.0	0.50	2.00122	2.00120	0.169344	0.169567	0.169545	0.017	1.25506	0.00053	0.8447	1.1090
37	15	0.991206	4.0	0.10	0.40025	3.60202	0.219202	0.219480	0.219467	0.011	0.94885	0.00023	0.8724	1.1308
38	15	0.991206	4.0	0.10	0.40021	3.60218	0.219197	0.219475	0.219462	0.011	0.94898	0.00023	0.8724	1.1308
39	15	0.991206	4.0	0.30	1.20070	2.80167	0.185714	0.185990	0.185977	0.011	1.07527	0.00026	0.8599	1.1199
40	15	0.991206	4.0	0.30	1.20067	2.80165	0.185710	0.185986	0.185973	0.011	1.07538	0.00026	0.8599	1.1199
41	15	0.991206	4.0	0.50	2.00127	2.00128	0.166328	0.166601	0.166588	0.011	1.23064	0.00029	0.8453	1.1069
42	15	0.991206	4.0	0.50	2.00122	2.00118	0.166330	0.166603	0.166590	0.011	1.23062	0.00029	0.8453	1.1069
37	20	0.990496	4.0	0.10	0.40025	3.60202	0.216810	0.217173	0.217133	0.011	0.93369	0.00034	0.8721	1.1284
38	20	0.990496	4.0	0.10	0.40021	3.60218	0.216803	0.217166	0.217126	0.011	0.93385	0.00034	0.8721	1.1284
39	20	0.990496	4.0	0.30	1.20070	2.80167	0.182792	0.183151	0.183112	0.011	1.05704	0.00038	0.8600	1.1175
40	20	0.990496	4.0	0.30	1.20067	2.80165	0.182788	0.183147	0.183108	0.011	1.05714	0.00038	0.8600	1.1175
41	20	0.990496	4.0	0.50	2.00127	2.00128	0.163193	0.163549	0.163509	0.011	1.20685	0.00044	0.8459	1.1047
42	20	0.990496	4.0	0.50	2.00122	2.00118	0.163190	0.163546	0.163506	0.011	1.20695	0.00044	0.8459	1.1047
37	25	0.991236	4.0	0.10	0.40025	3.60202	0.214281	0.214735	0.214672	0.021	0.91832	0.00038	0.8720	1.1258
38	25	0.991236	4.0	0.10	0.40021	3.60218	0.214274	0.214728	0.214665	0.021	0.91848	0.00038	0.8720	1.1258
39	25	0.991236	4.0	0.30	1.20070	2.80167	0.179769	0.180219	0.180155	0.020	1.03793	0.00041	0.8604	1.1151
40	25	0.991236	4.0	0.30	1.20067	2.80165	0.179770	0.180220	0.180156	0.020	1.03793	0.00041	0.8604	1.1151
41	25	0.991236	4.0	0.50	2.00127	2.00128	0.160008	0.160452	0.160389	0.054	1.18109	0.00124	0.8467	1.1024
42	25	0.991236	4.0	0.50	2.00122	2.00118	0.159887	0.160331	0.160268	0.054	1.18391	0.00125	0.8467	1.1024
43	30	0.989568	4.0	0.10	0.40022	3.60222	0.211535	0.212146	0.212053	0.021	0.90420	0.00042	0.8719	1.1232
44	30	0.989568	4.0	0.10	0.40017	3.60199	0.211529	0.212140	0.212047	0.021	0.90438	0.00042	0.8720	1.1231
45	30	0.989568	4.0	0.30	1.20075	2.80174	0.176553	0.177157	0.177065	0.039	1.01980	0.00080	0.8607	1.1127
46	30	0.989568	4.0	0.30	1.20071	2.80166	0.176562	0.177166	0.177074	0.039	1.01966	0.00080	0.8607	1.1127
48	30	0.989568	4.0	0.50	2.00122	2.00120	0.156465	0.157062	0.156970	0.122	1.16048	0.00273	0.8474	1.1001
43	40	0.990456	4.0	0.10	0.40022	3.60222	0.205718	0.206724	0.206689	0.040	0.87294	0.00087	0.8722	1.1177
44	40	0.990456	4.0	0.10	0.40017	3.60199	0.205712	0.206718	0.206683	0.040	0.87312	0.00087	0.8722	1.1177
45	40	0.990456	4.0	0.30	1.20075	2.80174	0.169780	0.170775	0.170740	0.075	0.98103	0.00151	0.8617	1.1076
46	40	0.990456	4.0	0.30	1.20071	2.80166	0.169915	0.170910	0.170875	0.075	0.97861	0.00151	0.8617	1.1076
48	40	0.990456	4.0	0.50	2.00122	2.00120	0.149187	0.150169	0.150135	0.075	1.11322	0.00172	0.8491	1.0954
63	5	0.991443	4.0	0.50	2.00125	2.00127	0.172286	0.172461	0.172465	0.014	1.27756	0.00045	0.8441	1.1110
64	5	0.991443	4.0	0.50	2.00143	2.00143	0.172289	0.172464	0.172468	0.014	1.27737	0.00045	0.8441	1.1110
63	10	0.989035	4.0	0.50	2.00125	2.00127	0.169342	0.169587	0.169566	0.017	1.25452	0.00053	0.8447	1.1090
64	10	0.989035	4.0	0.50	2.00143	2.00143	0.169344	0.169589	0.169568	0.017	1.25436	0.00053	0.8447	1.1090
67	10	1.012001	4.0	0.30	1.20079	2.80189	0.188788	0.188753	0.188731	0.070	1.09351	0.00159	0.8597	1.1221
68	10	1.012001	4.0	0.30	1.20086	2.80187	0.188794	0.188759	0.188737	0.070	1.09333	0.00159	0.8597	1.1221
63	15	0.990476	4.0	0.50	2.00125	2.00127	0.166322	0.166604	0.166591	0.011	1.23057	0.00029	0.8453	1.1069
64	15	0.990476	4.0	0.50	2.00143	2.00143	0.166325	0.166607	0.166594	0.011	1.23038	0.00029	0.8453	1.1069
63	20	1.001550	4.0	0.50	2.00125	2.00127	0.163325	0.163538	0.163498	0.011	1.20712	0.00044	0.8459	1.1047
64	20	1.001550	4.0	0.50	2.00143	2.00143	0.163329	0.163542	0.163502	0.011	1.20691	0.00044	0.8459	1.1047
63	25	1.001352	4.0	0.50	2.00125	2.00127	0.160085	0.160395	0.160332	0.054	1.18241	0.00125	0.8467	1.1024
64	25	1.001352	4.0	0.50	2.00143	2.00143	0.160088	0.160398	0.160335	0.054	1.18224	0.00125	0.8467	1.1025
63	30	1.000464	4.0	0.50	2.00125	2.00127	0.156673	0.157122	0.157030	0.122	1.15913	0.00272	0.8474	1.1001
64	30	1.000464	4.0	0.50	2.00143	2.00143	0.156671	0.157120	0.157028	0.122	1.15907	0.00272	0.8474	1.1002

67	30	1.009543	4.0	0.30	1.20079	2.80189	0.176884	0.177218	0.177126	0.039	1.01858	0.00080	0.8607	1.1127
68	30	1.009543	4.0	0.30	1.20086	2.80187	0.176889	0.177223	0.177131	0.039	1.01844	0.00080	0.8607	1.1127
63	40	1.001372	4.0	0.50	2.00125	2.00127	0.149473	0.150297	0.150263	0.075	1.11055	0.00171	0.8491	1.0954
64	40	1.001372	4.0	0.50	2.00143	2.00143	0.149466	0.150290	0.150256	0.075	1.11059	0.00171	0.8491	1.0954
67	40	1.008458	4.0	0.30	1.20079	2.80189	0.170161	0.170897	0.170863	0.075	0.97878	0.00151	0.8617	1.1076
68	40	1.008458	4.0	0.30	1.20086	2.80187	0.170165	0.170901	0.170867	0.075	0.97867	0.00151	0.8617	1.1076
49	5	0.999072	5.0	0.10	0.50031	4.50318	0.208348	0.208430	0.208434	0.147	1.07916	0.00332	0.8425	1.1569
50	5	0.999072	5.0	0.10	0.50037	4.50334	0.208044	0.208126	0.208130	0.147	1.08594	0.00334	0.8425	1.1569
51	5	0.999072	5.0	0.30	1.50108	3.50256	0.173956	0.174036	0.174040	0.011	1.27669	0.00039	0.8230	1.1454
52	5	0.999072	5.0	0.30	1.50112	3.50270	0.173962	0.174042	0.174046	0.011	1.27649	0.00039	0.8230	1.1454
53	5	0.999072	5.0	0.50	2.50185	2.50192	0.153134	0.153212	0.153216	0.012	1.52689	0.00049	0.7999	1.1313
54	5	0.999072	5.0	0.50	2.50197	2.50191	0.153126	0.153204	0.153208	0.012	1.52709	0.00049	0.7999	1.1313
55	10	0.989549	5.0	0.10	0.50040	4.50337	0.205659	0.205897	0.205875	0.095	1.06624	0.00209	0.8421	1.1543
56	10	0.989549	5.0	0.10	0.50036	4.50341	0.205656	0.205894	0.205872	0.095	1.06633	0.00209	0.8421	1.1543
57	10	0.989549	5.0	0.30	1.50115	3.50282	0.170982	0.171217	0.171195	0.055	1.25291	0.00144	0.8234	1.1429
58	10	0.989549	5.0	0.30	1.50114	3.50274	0.171112	0.171347	0.171325	0.055	1.24959	0.00144	0.8234	1.1429
59	10	0.989549	5.0	0.50	2.50189	2.50195	0.149974	0.150206	0.150184	0.159	1.49277	0.00488	0.8010	1.1289
60	10	0.989549	5.0	0.50	2.50184	2.50184	0.149658	0.149890	0.149868	0.159	1.50250	0.00492	0.8010	1.1289
49	15	0.996921	5.0	0.10	0.50031	4.50318	0.203483	0.203682	0.203669	0.106	1.04332	0.00223	0.8416	1.1515
50	15	0.996921	5.0	0.10	0.50037	4.50334	0.203233	0.203432	0.203419	0.106	1.04850	0.00224	0.8416	1.1515
51	15	0.996921	5.0	0.30	1.50108	3.50256	0.168086	0.168282	0.168268	0.010	1.22859	0.00027	0.8236	1.1403
52	15	0.996921	5.0	0.30	1.50112	3.50270	0.168088	0.168284	0.168270	0.010	1.22851	0.00027	0.8236	1.1403
53	15	0.996921	5.0	0.50	2.50185	2.50192	0.146847	0.147038	0.147025	0.096	1.45966	0.00283	0.8019	1.1265
54	15	0.996921	5.0	0.50	2.50197	2.50191	0.146713	0.146904	0.146891	0.096	1.46354	0.00283	0.8019	1.1265
49	20	0.996102	5.0	0.10	0.50031	4.50318	0.200808	0.201089	0.201049	0.096	1.02689	0.00198	0.8410	1.1487
50	20	0.996102	5.0	0.10	0.50037	4.50334	0.200613	0.200894	0.200854	0.096	1.03078	0.00198	0.8410	1.1487
51	20	0.996102	5.0	0.30	1.50108	3.50256	0.164996	0.165272	0.165232	0.010	1.20452	0.00042	0.8238	1.1376
52	20	0.996102	5.0	0.30	1.50112	3.50270	0.164997	0.165273	0.165233	0.010	1.20446	0.00042	0.8238	1.1376
53	20	0.996102	5.0	0.50	2.50185	2.50192	0.143523	0.143793	0.143753	0.094	1.42729	0.00269	0.8028	1.1240
54	20	0.996102	5.0	0.50	2.50197	2.50191	0.143391	0.143661	0.143621	0.094	1.43097	0.00270	0.8028	1.1240
49	25	0.996062	5.0	0.10	0.50031	4.50318	0.198004	0.198381	0.198317	0.059	1.00991	0.00116	0.8409	1.1458
50	25	0.996062	5.0	0.10	0.50037	4.50334	0.197998	0.198375	0.198311	0.059	1.00995	0.00116	0.8409	1.1458
51	25	0.996062	5.0	0.30	1.50108	3.50256	0.161822	0.162192	0.162129	0.020	1.17910	0.00046	0.8244	1.1349
52	25	0.996062	5.0	0.30	1.50112	3.50270	0.161818	0.162188	0.162125	0.020	1.17916	0.00046	0.8244	1.1349
53	25	0.996062	5.0	0.50	2.50185	2.50192	0.140120	0.140482	0.140419	0.107	1.39350	0.00291	0.8040	1.1215
54	25	0.996062	5.0	0.50	2.50197	2.50191	0.139971	0.140333	0.140270	0.107	1.39749	0.00291	0.8040	1.1215
55	30	0.988552	5.0	0.10	0.50040	4.50337	0.195059	0.195665	0.195573	0.052	0.99142	0.00101	0.8408	1.1429
56	30	0.988552	5.0	0.10	0.50036	4.50341	0.195052	0.195658	0.195566	0.052	0.99158	0.00101	0.8408	1.1429
57	30	0.988552	5.0	0.30	1.50115	3.50282	0.158452	0.159050	0.158957	0.024	1.15361	0.00060	0.8249	1.1322
58	30	0.988552	5.0	0.30	1.50114	3.50274	0.158459	0.159056	0.158964	0.024	1.15346	0.00060	0.8249	1.1322
59	30	0.988552	5.0	0.50	2.50189	2.50195	0.136404	0.136991	0.136898	0.110	1.36302	0.00289	0.8052	1.1190
60	30	0.988552	5.0	0.50	2.50184	2.50184	0.136189	0.136776	0.136683	0.110	1.36868	0.00290	0.8052	1.1190
55	40	0.988246	5.0	0.10	0.50040	4.50337	0.188944	0.189949	0.189914	0.280	0.95275	0.00500	0.8413	1.1369
56	40	0.988246	5.0	0.10	0.50036	4.50341	0.188947	0.189952	0.189917	0.280	0.95273	0.00500	0.8413	1.1369
57	40	0.988246	5.0	0.30	1.50115	3.50282	0.151376	0.152365	0.152331	0.082	1.10368	0.00183	0.8266	1.1267
58	40	0.988246	5.0	0.30	1.50114	3.50274	0.151486	0.152476	0.152441	0.082	1.10144	0.00183	0.8266	1.1267
59	40	0.988246	5.0	0.50	2.50189	2.50195	0.128334	0.129304	0.129270	0.243	1.31068	0.00598	0.8079	1.1139
60	40	0.988246	5.0	0.50	2.50184	2.50184	0.128649	0.129619	0.129585	0.243	1.30309	0.00594	0.8079	1.1139
65	5	0.991443	5.0	0.10	0.50044	4.50392	0.208243	0.208418	0.208421	0.147	1.07921	0.00332	0.8425	1.1569
66	5	0.991443	5.0	0.10	0.50048	4.50376	0.208237	0.208412	0.208415	0.147	1.07930	0.00332	0.8425	1.1569
65	10	0.989035	5.0	0.10	0.50044	4.50392	0.205816	0.206060	0.206039	0.095	1.06256	0.00209	0.8421	1.1543
66	10	0.989035	5.0	0.10	0.50048	4.50376	0.205811	0.206055	0.206034	0.095	1.06263	0.00209	0.8421	1.1543
69	10	1.012001	5.0	0.30	1.50140	3.50306	0.171309	0.171268	0.171246	0.055	1.25144	0.00144	0.8234	1.1429
70	10	1.012001	5.0	0.30	1.50127	3.50292	0.171315	0.171274	0.171252	0.055	1.25137	0.00144	0.8234	1.1429

71	10	1.012001	5.0	0.50	2.50215	2.50210	0.149899	0.149855	0.149833	0.159	1.50341	0.00492	0.8009	1.1289
72	10	1.012001	5.0	0.50	2.50217	2.50212	0.150070	0.150026	0.150004	0.159	1.49814	0.00490	0.8009	1.1289
65	15	0.990476	5.0	0.10	0.50044	4.50392	0.203327	0.203608	0.203595	0.106	1.04466	0.00223	0.8416	1.1515
66	15	0.990476	5.0	0.10	0.50048	4.50376	0.203325	0.203606	0.203593	0.106	1.04467	0.00223	0.8416	1.1515
65	20	1.001550	5.0	0.10	0.50044	4.50392	0.200873	0.201084	0.201044	0.096	1.02678	0.00198	0.8410	1.1487
66	20	1.001550	5.0	0.10	0.50048	4.50376	0.200871	0.201082	0.201042	0.096	1.02678	0.00198	0.8410	1.1487
65	25	1.001352	5.0	0.10	0.50044	4.50392	0.198167	0.198474	0.198411	0.059	1.00787	0.00116	0.8409	1.1458
66	25	1.001352	5.0	0.10	0.50048	4.50376	0.198169	0.198476	0.198413	0.059	1.00779	0.00116	0.8409	1.1458
65	30	1.000464	5.0	0.10	0.50044	4.50392	0.195299	0.195743	0.195651	0.052	0.98984	0.00101	0.8408	1.1429
66	30	1.000464	5.0	0.10	0.50048	4.50376	0.195302	0.195746	0.195654	0.052	0.98975	0.00101	0.8408	1.1429
69	30	1.009543	5.0	0.30	1.50140	3.50306	0.158759	0.159073	0.158980	0.024	1.15294	0.00060	0.8249	1.1322
70	30	1.009543	5.0	0.30	1.50127	3.50292	0.158763	0.159077	0.158984	0.024	1.15294	0.00060	0.8249	1.1322
72	30	1.009543	5.0	0.50	2.50217	2.50212	0.136607	0.136910	0.136818	0.110	1.36499	0.00289	0.8052	1.1190
65	40	1.001372	5.0	0.10	0.50044	4.50392	0.188676	0.189491	0.189457	0.280	0.96076	0.00504	0.8413	1.1370
66	40	1.001372	5.0	0.10	0.50048	4.50376	0.189326	0.190141	0.190107	0.280	0.94923	0.00498	0.8413	1.1370
69	40	1.008458	5.0	0.30	1.50140	3.50306	0.151652	0.152351	0.152317	0.082	1.10383	0.00183	0.8265	1.1267
70	40	1.008458	5.0	0.30	1.50127	3.50292	0.151790	0.152489	0.152455	0.082	1.10108	0.00183	0.8266	1.1267
72	40	1.008458	5.0	0.50	2.50217	2.50212	0.129094	0.129774	0.129740	0.243	1.29919	0.00593	0.8079	1.1139

[a] Columns mCl$^-$ and yH$^+$ contain rounded values, and exact values can be calculated from the listed *m*HCl and *m*TrisHCl.

[b] Measurement temperatures are equal to 5.000 °C, 10.000 °C, etc. within the expanded uncertainty of the thermometer (±0.007 °C, k = 2 from the calibration certificate), and are presented here as integer values.

[c] The meanings of the listed potentials are as follows: *E*(meas.) – the unadjusted measured values at the listed pressure, *P*; *E* – measured potentials corrected to 1 atm pH$_2$ (and with results for cells 61 and 62 adjusted by adding -0.0001289 V as described in the notes to Table 5); *E*(adj.) – values of *E* adjusted to the standard potentials of Bates and Bower,[12] as described in section 3.1 of the main text.

References

(1) I. Maksimov, T. Asakai, and M. Ohata (2023) On the optimal use of silver–silver chloride reference electrodes. *Accreditation and Quality Assurance* **28**, 65-68; doi.org/10.1007/s00769-023-01533-2

(2) R. G. Bates *Determination of pH, Theory and Practice*. Wiley, New York, 1973, 496pp.

(3) Joint Committee for Guides in Metrology (2008) *Evaluation of measurement data — Guide to the expression of uncertainty in measurement*. BIPM, IEC, IFCC, ILAC, ISO, IUPAC, IUPAP and OIML, 120 pp, doi.org/10.59161/JCGM100-2008E.

(4) G. S. Kell (1975) Density, thermal expansibility and compressibility of liquid water from 0 to 150 °C: corrections and tables for atmospheric pressure and saturation reviewed and expressed on 1968 temperature scale. *J. Chem. Eng. Data* **20**, 97-105.

(5) S. L. Clegg and A. S. Wexler (2011) Densities and apparent molar volumes of atmospherically important electrolyte solutions. I. The solutes H_2SO_4, HNO_3, HCl, Na_2SO_4, $NaNO_3$, NaCl, $(NH_4)_2SO_4$, NH_4NO_3, and NH_4Cl from 0 to 50 °C, including extrapolations to very low temperature and to the pure liquid state, and $NaHSO_4$, NaOH and NH_3 at 25 °C. *J. Phys. Chem. A* **115**, 3393-3460.

(6) T. D. Ford, T. G. Call, M. L. Origlia, M. A. Stark, and E. M. Woolley (2000) Apparent molar volumes and apparent molar heat capacities of aqueous 2-amino-2-hydroxymethyl-propan-1,3-diol (Tris or THAM) and THAM plus equimolal HCl. *J. Chem. Thermodyn.* **32**, 499-516.

(7) P. Ya. Tishchenko (2000) Non-ideal properties of the TRIS-TRIS.HCI-NaCI-H_2O buffer system in the 0-40 °C temperature interval. Application of the Pitzer equations. *Russ. Chem. Bull.* **49**, 4, 674-679.

(8) H. F. Holmes, R. H. Busey, J. M. Simonson, R. E. Mesmer, D. G. Archer, and R. H. Wood (1987) The enthalpy of dilution of HCl(aq) to 648 K and 40 MPa. Thermodynamic properties. *J. Chem. Thermo.* **19**, 863-890.

(9) R. G. Bates and J. B. Macaskill (1985) Activity and osmotic coefficients of t-butylammonium chloride: activity of HCl in mixtures with TRIS hydrochloride and t-butylammonium chloride at 25 °C. *J. Solut. Chem.* **14**, 723-734.

(10) S. L. Clegg, M. P. Humphreys, J. F. Waters, D. R. Turner, and A. G. Dickson (2022) Chemical speciation models based upon the Pitzer activity coefficient equations, including the propagation of uncertainties. II. Tris buffers in artificial seawater at 25 °C, and an assessment of the seawater 'Total' pH scale. *Mar. Chem.* **244**, art. no. 104096.

(11) P. Brimblecombe and S. L. Clegg (1988) The solubility and behaviour of acid gases in the marine aerosol. *J. Atmos. Chem.* **7**, 1-18.

(12) R. G. Bates and V. E. Bower (1954) Standard potential of the silver-silver chloride electrode from 0 to 95 °C and the thermodynamic properties of dilute hydrochloric acid solutions. *J. Res. Natl. Bur. Stnds.* **53**, 283-290.
